# Phospholipid scramblase 1: an essential component of the nephrocyte slit diaphragm

**DOI:** 10.1007/s00018-024-05287-z

**Published:** 2024-06-15

**Authors:** Vicente Castillo-Mancho, Alexandra Atienza-Manuel, Jorge Sarmiento-Jiménez, Mar Ruiz-Gómez, Joaquim Culi

**Affiliations:** Centro de Biología Molecular Severo Ochoa, CSIC and UAM, Nicolás Cabrera 1, Cantoblanco, Madrid, 28049 Spain

**Keywords:** Slit diaphragm, Intercellular junction, Membrane dynamics

## Abstract

Blood ultrafiltration in nephrons critically depends on specialized intercellular junctions between podocytes, named slit diaphragms (SDs). Here, by studying a homologous structure found in *Drosophila* nephrocytes, we identify the phospholipid scramblase Scramb1 as an essential component of the SD, uncovering a novel link between membrane dynamics and SD formation. In *scramb1* mutants, SDs fail to form. Instead, the SD components Sticks and stones/nephrin, Polychaetoid/ZO-1, and the Src-kinase Src64B/Fyn associate in cortical foci lacking the key SD protein Dumbfounded/NEPH1. Scramb1 interaction with Polychaetoid/ZO-1 and Flotillin2, the presence of essential putative palmitoylation sites and its capacity to oligomerize, suggest a function in promoting SD assembly within lipid raft microdomains. Furthermore, Scramb1 interactors as well as its functional sensitivity to temperature, suggest an active involvement in membrane remodeling processes during SD assembly. Remarkably, putative Ca^2+^-binding sites in Scramb1 are essential for its activity raising the possibility that Ca^2+^ signaling may control the assembly of SDs by impacting on Scramb1 activity.

**Supplementary Information:**

The online version contains supplementary material available at 10.1007/s00018-024-05287-z.

## Introduction

The role of the kidneys in maintaining body homeostasis stems from the vital process of glomerular ultrafiltration, which results in the formation of the primary urine. Filtration occurs through two cellular layers: the fenestrated capillary endothelium and a sheet composed of podocytes, and their shared basal membrane. Numerous projections emanating from podocytes intertwine with each other to completely cover the glomerular capillaries. These projections are held together by a specialized intercellular junction named the slit diaphragm (SD), forming a continuous structure resembling a zipper that acts as a molecular filter. Mutations in many of the SD components cause the loss of these junctions, the development of congenital nephrotic syndromes, and failures in the renal function associated with proteinuria [1–3]. The main constituents of the SD are nephrin and NEPH1, which are transmembrane adhesion molecules of the immunoglobulin superfamily. Their extracellular regions contribute to the formation of the sieves [4–8], whereas their cytoplasmic regions provide association with other components of the SD complex such as CD2AP and ZO-1, participating in signaling hubs that regulate many aspects of the podocyte biology [9].

*Drosophila* nephrocytes, cells of the insect excretory system involved in hemolymph ultrafiltration, have SDs structurally, molecularly, and functionally analogous to the podocyte SDs. The entire nephrocyte surface contains deep membrane invaginations -the labyrinthine channels- that are sealed from the surrounding hemolymph by SDs, which are arranged in long strands depicting a fingerprint-like pattern. Sticks and stones (Sns) and Dumbfounded (Duf, also referred as Kin of irre, Kirre), orthologs to nephrin and NEPH1 respectively, interact through their extracellular domains to form these junctions [10–13].

Podocyte SDs are harbored in cholesterol-rich lipid raft microdomains of the plasma membrane. This lipid environment is essential for the assembly, stability, and signaling of SDs [14–21]. The role of lipids in *Drosophila* SD dynamics is less well known. However, recent findings showed that recycling of *Drosophila* SD proteins partly depends on a process of raft-mediated endocytosis and is affected by depletion of cholesterol from membranes [22]. In addition, the phospholipid PI(4,5)P2 is essential for SD formation and plays a key role in defining membrane domains prone to the stabilization of SD components, where the first SDs are assembled during embryogenesis [23, 24]. Thus, membrane lipid composition significantly impacts multiple aspects of SD biology. In this context, we were intrigued by the specific expression of the gene *scramblase 1* (*scramb1*) in embryonic *Drosophila* nephrocytes [25]. Scramb1 has homology to human Phospholipid Scramblases, a family of proteins that associate to membranes and promote the bidirectional translocation of phospholipid across the two membrane leaflets in vitro and in vivo [26–32, 59]. The enrichment of Scramb1 in nephrocytes pointed to a possible involvement of this protein in the formation and function of SDs.

Here we demonstrate that Scramb1 localizes to SDs and is essential for their formation. Our data support a model in which Scramb1 associates with plasma membrane regions enriched in lipid raft microdomains, where it plays a scaffolding function promoting SD assembly. This is facilitated by Scramb1 palmitoylation, its interaction with Polychaetoid (Pyd), the *Drosophila* ZO-1 ortholog, and its capacity to self-oligomerize. Moreover, Scramb1 physical and genetic interactions with membrane remodeling proteins suggest an active role in membrane remodeling processes occurring during SD assembly.

## Results

### Scramb1 is a novel constituent of the *Drosophila* Slit Diaphragm

**Fig. 1:**
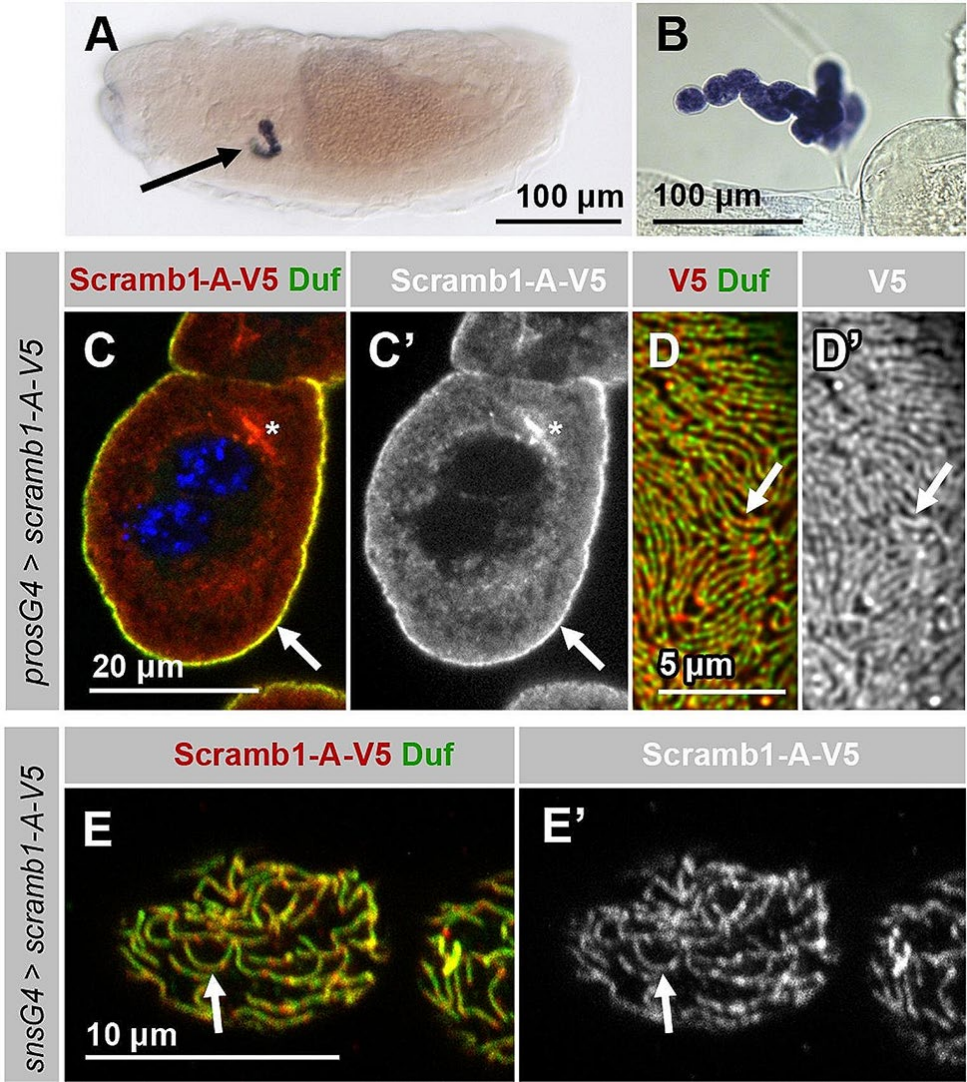
*scramb1* expression and subcellular localization. **(A-B) ***scramb1* is expressed in the garland nephrocytes of stage 15 *Drosophila* embryos (A, arrow) and of third instar larvae (B) as detected by in situ hybridization. **(C-E’)** Distribution of Scramb1-A-V5 driven by the indicated *Gal4* lines (anti-V5 antibody) and Duf in garland nephrocytes of third instar (C-D’) and first instar (E-E’) larvae shown at a medial section (C-C’) and at higher magnifications at cortical levels (D-E’). Scramb1-A-V5 colocalizes with Duf in SDs (arrows). Asterisks in C and C’ point to cytoplasmic aggregates. (C) Nuclei were stained with DAPI (blue)

Phospholipid scramblase *scramb1* is initially expressed in the subesophageal region of stage 12 embryos, where garland nephrocytes are located. Its expression becomes more robust in later stages of embryogenesis (Fig. 1A and Fig. S1 A). Throughout larval stages, *scramb1* continues to be expressed in garland nephrocytes and it is activated in additional tissues, including pericardial cells, the Malpighian tubules and the neuromuscular synapsis (Fig. 1B; Fig. S1 B and [33, 34]).

*scramb1* is transcribed from two alternative promoters, generating two isoforms, Scramb1-A and -B. Scramb1-A, the predominant isoform in nephrocytes according to RNAseq data (Fig. S1, C and F; and Methods section), is longer due to the inclusion of an N-terminal proline-rich region.

To examine Scramb1 subcellular distribution we generated a V5-tagged version of Scramb1-A and expressed it in nephrocytes. The protein accumulates at the cortical region (Fig. 1, C and C’, arrows), drawing a distinctive fingerprint-like pattern that corresponds to SDs, as revealed by its colocalization with Duf (Fig. 1D-E’, arrows). A cytoplasmic distribution and occasional cytoplasmic aggregates are also observed, possibly caused by non-physiological overexpression conditions (Fig. 1C and C’, asterisks).

Together, these findings indicate that *scramb1* is highly expressed in nephrocytes and that the protein localizes within SDs, suggesting it could be a novel component of the SD protein complex.

### *scramb1* is required for SDs formation

**Fig. 2:**
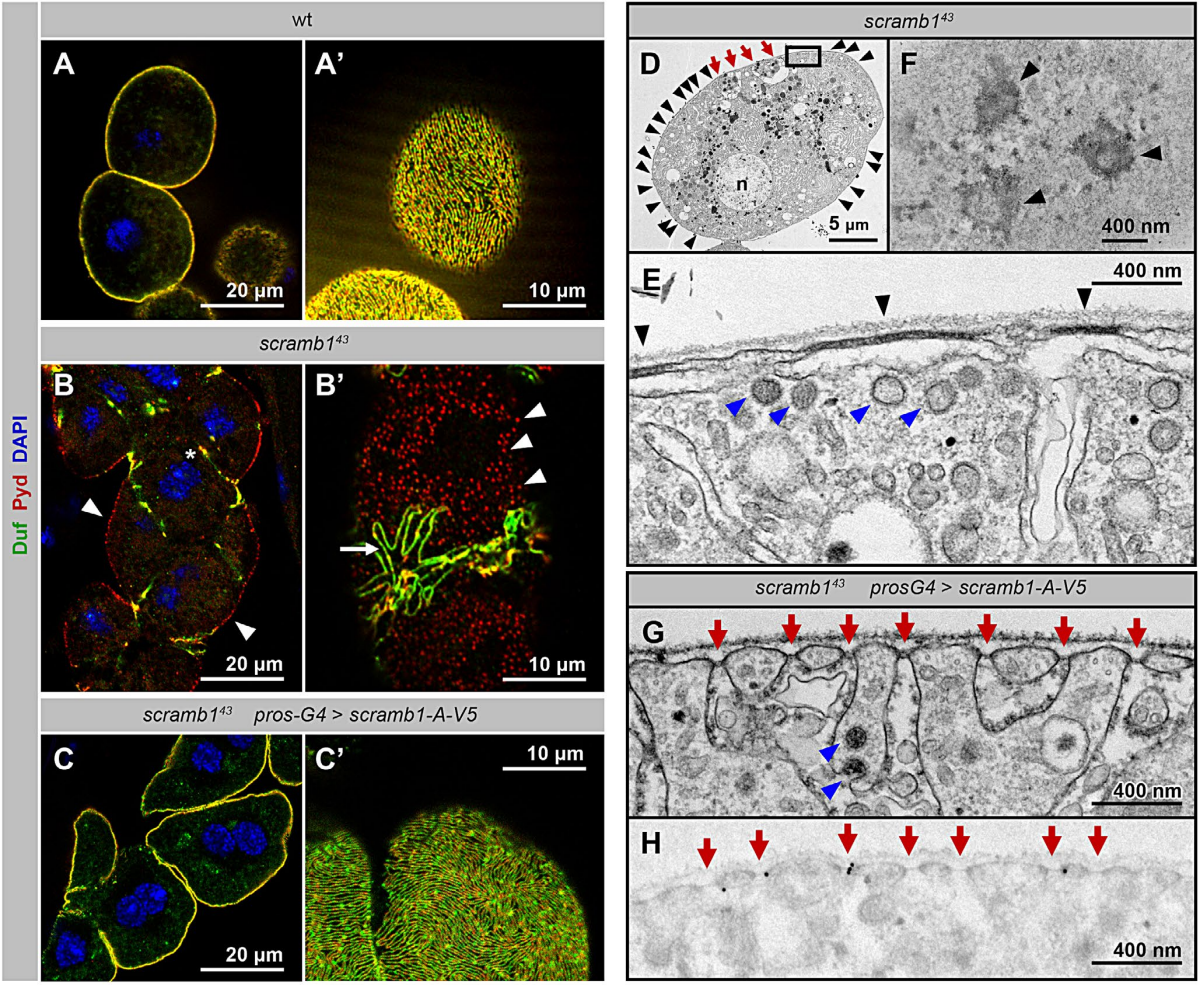
*scramb1* loss of function phenotype. **(A-B’)** Immunostaining of wild-type (A-A’) and *scramb1*^*43*^ (B-B’) garland nephrocytes depicting the distribution of the SD proteins Duf and Pyd. In *scramb1*^*43*^ nephrocytes, SD strands are sparse (arrow, expressing Duf and Pyd). Pyd predominantly accumulates in cortical foci devoid of Duf (arrowheads). Furthermore, Duf and Pyd colocalize in certain regions of contact between clustered nephrocytes (asterisk). **(C-C’)** Immunostaining of *scramb1*^*43*^ larval nephrocytes phenotypically rescued by the expression of *UAS-scramb1-A-V5* driven by *pros-Gal4*, to show the expression of Duf and Pyd, as indicated. SD strands cover the entire nephrocyte surface. (A, B and C) medial planes. (A’, B’ and C’) cortical planes. **(D-F)** TEM images of *scramb1*^*43*^ nephrocytes. An overview of a complete nephrocyte is shown in D (n: nucleus). The highlighted region is shown at higher magnification in E. Electron-dense plaques (black arrowheads in D and E) that bridge the plasma membrane with sub-cortical lacunae are frequently observed. (F) Tangential section through the nephrocyte cortex displaying electron-dense circular structures (black arrowheads) that might correspond to the cortical electron-dense plaques observed in cross-sections. Occasional SDs are also observed (D, red arrows. See also Fig. S2 G). **(G)** TEM image of a *scramb1*^*43*^ mutant nephrocyte rescued by the expression of *UAS-scramb1-A-V5*, displaying a normal density of SDs (red arrows). Blue arrowheads in E and G point to clathrin coated vesicles and pits. **(H)** Immunogold labelling of Scramb1-A-V5 (anti-V5 antibody) in a nephrocyte of the same genotype as in G, showing that gold particles associate with SDs (red arrows). Statistical analysis described in the Methods section

To examine *scramb1* function in nephrocytes, we generated novel loss-of-function alleles by the imprecise excision of a P-element inserted in the first intron of the gene (*scramb1*^*EY07744*^). In *scramb1*^*43*^, a deletion of 2.9 kb of genomic DNA removes the transcription start sites of all *scramb1* isoforms, resulting in a probable null allele. Accordingly, transcripts could not be detected either by RT-PCR or by in situ hybridization (Fig. S1, C-E). *scramb1*^*43*^ mutant flies are homozygous viable and fertile, and they do not exhibit any visible macroscopic phenotype, which is consistent with previous reports for another *scramb1* allele [33]. In contrast, the larval garland nephrocytes show gross morphological abnormalities. Instead of displaying its distinctive garland-like cellular arrangement, nephrocytes are aggregated in *scramb1*^*43*^, a phenotype characteristic of mutations that disrupt SD formation (Fig. 2B, compare with wild-type in A). Accordingly, *scramb1*^*43*^ nephrocytes display either no SD strands (25% of nephrocytes) or only a few SD strands (75%) on their surface, mainly near regions of cell contact (*n* = 110 cells examined; Fig. 2, B and B’, arrows), contrasting with the multitude of SDs that in the wild type describe a dense fingerprint-like pattern on the nephrocyte surface (Fig. 2A’). Interestingly, broad regions of the plasma membrane of mutant nephrocytes are covered by foci of about 400 nm in diameter that accumulate Pyd but not Duf (Fig. 2, B and B’, arrowheads). In addition, Duf and Pyd are coexpressed in some regions of contact between the aggregated nephrocytes (Fig. 2B, asterisk). The described phenotypes are already apparent in first instar larvae, suggesting they are not caused by a possible degeneration of the nephrocytes (Fig. S2 B, compare with wild-type in A). Thus, our data indicate that *scramb1*^*43*^ nephrocytes are mostly devoid of SDs.

The presence of occasional SD-like structures in *scramb1*^*43*^ nephrocytes prompted us to examine whether *scramb2*, a *scramb1* paralog enriched in nephrocytes and displaying redundant activity with *scramb1* during synaptic transmission (Fig. S1 G, arrow and [33]), may also contribute to SD formation. To this end, we generated novel *scramb2* mutations using CRISPR-Cas9 genome-editing technology. *scramb2*^*V3*^, resulting in a frameshift mutation after Asp-16 and truncating 94% of the protein residues (Fig. S1 F), did not exhibit a discernible SD phenotype (Fig. S2, C and C’). In addition, a double mutant *scramb1*^*43*^, *scramb2*^*V6*^, an allele that produces a comparable Scramb2 truncation (Fig. S1 F), displayed a nephrocyte phenotype indistinguishable from that of *scramb1*^*43*^ (Fig. S2, D and D’, compare with Fig. 2, B and B’). Notably, while the expression of *UAS-scramb1-A-V5* in a *scramb1*^*43*^ mutant background fully restores SD formation, the expression of *UAS-scramb2-HA* showed no observable effect (Fig. 2, C and C’; and Fig. S2, E and E’). These findings indicate lack of functional redundancy between the two paralogs in nephrocytes.

As expected from the previous results, examination of the ultrastructure of *scramb1*^*43*^ nephrocytes by transmission electron microscopy (TEM) showed an almost complete absence of SDs in the plasma membrane (Fig. 2, D and E; quantitated in Fig. S2 H). This contrasted with the abundant SDs decorating the cortex in the wild-type (Fig. S2 F, arrows; Fig. S2 H; and [10, 11]) and in *scramb1*^*43*^ nephrocytes rescued by the expression of *UAS-scramb1-A-V5* (Fig. 2G, red arrows; quantitated in Fig. S2 H). Seldom, we observed structures similar to SDs and that probably correspond to the scarce SD-like strands observed by confocal microscopy (Fig. 2D and Fig. S2 G, red arrows; quantitated in Fig. S2 H). A remarkable characteristic of *scramb1*^*43*^ mutants is the presence of cisternae that run parallel to the plasma membrane beneath broad regions of electron-dense plaques of about 300–800 nanometers in length and that are absent in the wild-type or in rescued nephrocytes (Fig. 2, D and E, arrowheads; quantitated in Fig. S2 H). Circular electron-dense patches that might correspond to these plaques are occasionally visible in cortical tangential sections (Fig. 2F, arrowheads). These plaques could correspond to the plasma membrane foci that accumulate Pyd in *scramb1*^*43*^ nephrocytes observed by confocal microscopy, since their respective sizes and frequencies are compatible (Fig. 2B’, arrowheads). Similarly to the wild-type, abundant clathrin coated vesicles and pits are observed in cell from both *scramb1*^*43*^ mutants and rescued animals (Fig. 2, E and G, blue arrowheads), and the remaining ultrastructural organization of the nephrocytes remains largely unaffected.

Next, we examined the distribution of Scramb1-A by immunoelectron microscopy using anti-V5 antibodies in *scramb1*^*43*^ nephrocytes rescued by *UAS-scramb1-A-V5* expression. To reduce the accumulation of ectopic protein, we switched off *UAS-scramb1-A-V5* expression 72 h before fixation. This condition resulted in a complete rescue of SDs (Fig. 2G; quantitated in Fig. S2 H) and in Scramb1-A-V5 mostly located in the cortex, as visualized by confocal microscopy (Fig. S2 I). Notably, Scramb1-A-V5 signal was associated with SDs with a highly statistically significant value (see the Methods section), whereas no other structure was consistently labeled (Fig. 2H, red arrows point to SDs). These results demonstrate that Scramb1 is a constituent of the SD complex in *Drosophila* and that it is required for its assembly and/or maintenance.

### *scramb1* is required to recruit Duf to complexes containing Sns, Pyd and Src64B

We have shown that in the absence of *scramb1*, nephrocytes are largely devoid of SDs. *scramb1* might play a role either in the initial assembly of the SD complexes or in their subsequent maturation and maintenance. To better define *scramb1* role, we characterized the *de novo* formation of SDs by inducing the expression of Scramb1-A-V5 in *scramb1*^*43*^ nephrocytes during the larval stages using the TARGET technology, which allows the activation of transgenes through a temperature switch [35].

**Fig. 3:**
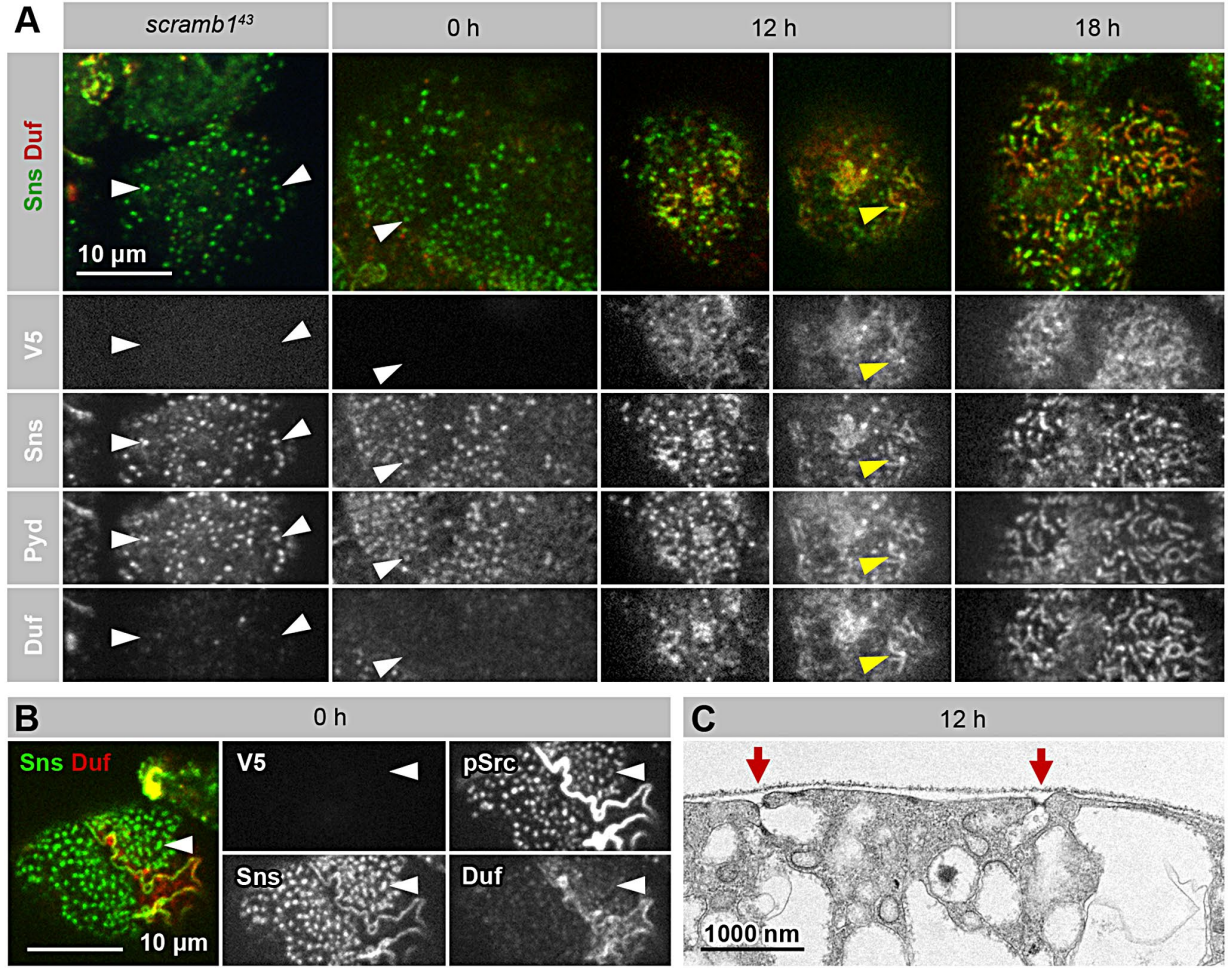
Time-course analysis of the induction of SD formation by Scramb1-A. **(A-B)** Immunostaining of *scramb1*^*43*^ or *scramb1*^*43*^ nephrocytes rescued by the expression of *UAS-scramb1-A-V5* for increasing periods of time (0, 12 and 18 h, as indicated) using the TARGET technology. See the Methods section for the complete genotype. The distribution of Scramb1-A-V5 (anti-V5 antibody), Sns, Pyd, Duf and phospho-Src64B in the cortical region are shown, as indicated. Each image corresponds to a Z-projection of several cortical planes. No SD strands are observed in nephrocytes that do not express *UAS-scramb1-A-V5*. Instead, abundant cortical foci containing Pyd, Sns and phospho-Src64B cover the nephrocyte surface (white arrowheads). At the 12 h window (two examples shown), Duf is visible in those foci and some acquire an elongated shape (yellow arrowheads). At 18 h, multiple short SD strands cover the surface of the nephrocytes. All images shown at the same magnification. See Fig. S3 for additional time points and medial sections. **(C)** TEM image of a *scramb1*^*43*^ nephrocyte rescued by the expression of *UAS-scramb1-A-V5* for 12 h. Multiple SDs sealing small labyrinthine channels are visible (red arrows). Electron-dense plaques, marked by black arrowheads in Fig. 2E, are rare

Similarly to *scramb1*^*43*^ mutants, at time zero before the expression of Scramb1-A-V5; Pyd and Sns colocalize in foci covering large regions of the nephrocyte plasma membrane (Fig. 3A and Fig. S3, white arrowheads). In contrast, Duf, an essential component of the SDs, is absent from these foci. We interpret these foci as representing aberrant pre-SDs complexes that cannot progress to form SDs in the absence of Scramb1 and in particular, cannot recruit Duf. This suggestion gains support from the observation that the kinase Src64B, the *Drosophila* ortholog of Fyn involved in SD formation and repair [12], is specifically active within the majority of these foci, as evidenced by phospho-Src64B accumulation (Fig. 3B, white arrowheads).

Scramb1-A-V5 protein becomes detectable in nephrocytes six hours after switching to the permissive temperature. At this time window, the cortical Pyd foci maintain the same morphology but begin to accumulate low levels of Scramb1-A-V5 and Duf (Fig. S3). After six additional hours (12-hour time point), short rods containing Sns, Duf, Pyd and phospho-Src64B can be observed on the surface of nephrocytes (Fig. 3A and Fig. S3, yellow arrowheads), along with circular foci displaying the same set of proteins (Fig. 3A and Fig. S3). Thus, Scramb1-A-V5 is recruited to pre-existing Sns/Pyd/Src64B-containing foci, along with Duf, and is necessary to allow the formation of elongated structures resembling the SD strands observed in the wild-type, albeit shorter. Supporting this interpretation, after 12 h of supplying Scramb1-A-V5, ultrastructural analyses revealed the presence of multiple SDs, usually associated to short labyrinthine channels. Interestingly, the electron-dense plaques characteristic of the *scramb1*^*43*^ mutant were almost absent at this time point (Fig. 3C, compare with Fig. 2E). Finally, 18 h after the temperature switch, most nephrocytes were covered by a loose network of strands containing Duf, Sns, Pyd and Scramb1-A-V5, similar to the SD fingerprint pattern observed in the wild-type but less regular and dense, indicating an already significant rescue of the phenotype (Fig. 3A).

These findings suggest that the inability to form SDs in the absence of Scramb1 is due, at least in part, to Duf not being recruited and stabilized in complexes that already contain other essential components of the SD, namely Sns, Pyd and phospho-Src64B. Duf recruitment is likely not mediated through a direct interaction with Scramb1, since Duf is unable to recruit Scramb1 in S2 cells (Fig. S4 A, arrows), indicating the necessity of additional factors absent in this cell line.

### The N-terminal proline-rich domain of Scramb1-A protects it from degradation and mediates its association with Pyd

**Fig. 4:**
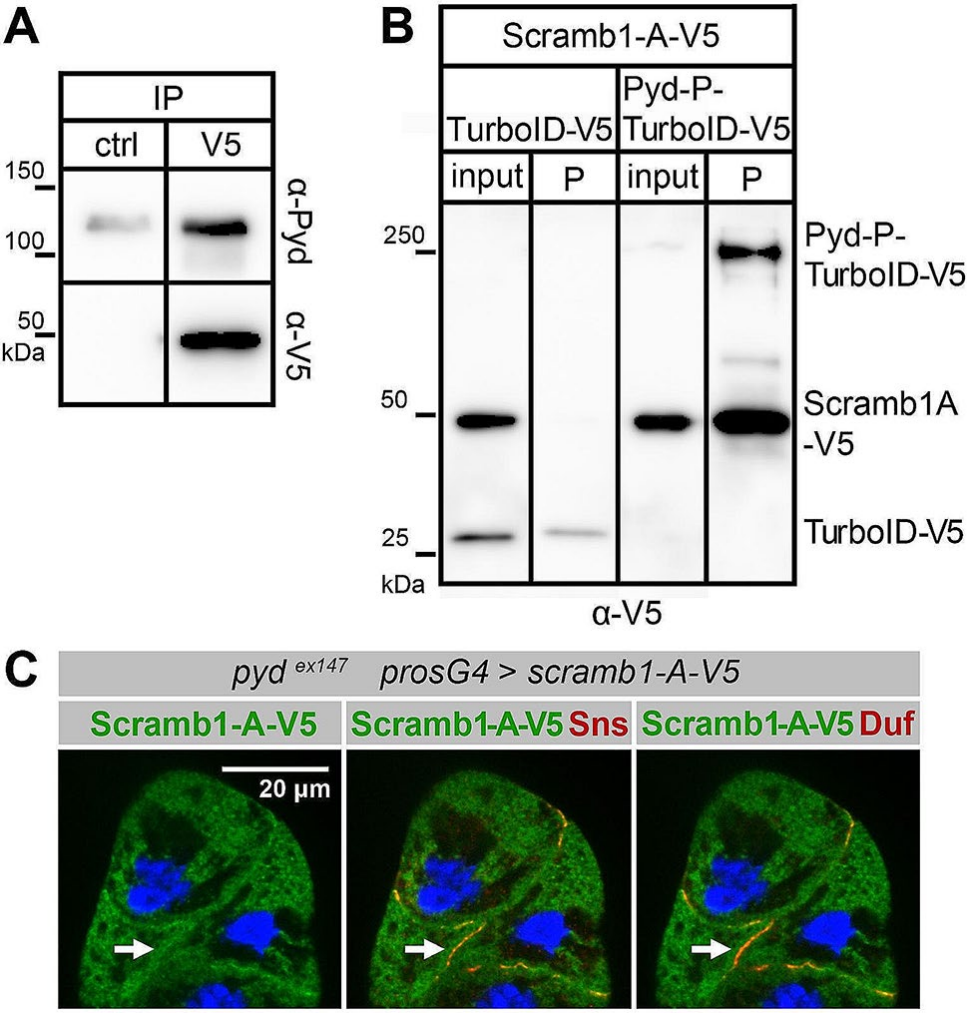
Scramb1-A interacts with Pyd. **(A)** Co-IP of Scramb1-A-V5 and Pyd-PΔCC from a lysate of salivary glands coexpressing both proteins. The same lysate was incubated with a magnetic matrix coupled to either anti-β-galactosidase in the control (ctrl) experiment or anti-V5 (V5) antibodies. The eluates were analyzed by western blot using anti-V5 to detect Scramb1-A-V5 and anti-Pyd. Pyd-PΔCC was notably elevated in the eluate from the V5 matrix compared to the control, which shows some unspecific Pyd binding to the matrix. **(B)** Proximity labeling with biotin of Scramb1-A-V5 by Pyd-P-TurboID-V5. Biotinylated proteins were isolated from lysates of salivary glands expressing Scramb1-A-V5 alongside Pyd-P-TurboID-V5 or TurboID-V5 (control). The lysates (input, 10% loaded) and purified fractions (P) were analyzed by western blot using anti-V5 antibody to detect TurboID-V5, Pyd-P-TurboID-V5 and Scramb1-A-V5. Scramb1-A-V5 was biotinylated by Pyd-P-TurboID-V5 but not by the control TurboID-V5, indicating a close association between Scramb1-A and Pyd. Notice that Pyd-P-TurboID-V5 and TurboID-V5 auto-biotinylate themselves. **(C)** Immunostaining of *pyd*^*ex147*^ nephrocytes expressing *UAS-scramb1-A-V5* driven by *pros-Gal4* to detect Scramb1-A-V5 (anti-V5 antibody), Sns and Duf, as indicated. Nuclei were labeled with DAPI (blue). *pyd*^*ex147*^ nephrocytes lack SDs and both Sns and Duf accumulate in regions of contact between aggregated nephrocytes (arrows) whereas Scramb1-A-V5 shows a cytoplasmic distribution

Scramb1 localization within SDs could be mediated by its interaction with other SD components. One potential candidate is Pyd, a scaffolding protein that contributes to link the SD complex to the cytoskeleton [36]. Noteworthy, Pyd contains an SH3 domain that could potentially bind to an unstructured, proline-rich region located at the N-terminus of the Scramb1-A isoform (Fig. S1 F). To test this hypothesis, we conducted co-immunoprecipitation assays using extracts from salivary glands expressing both proteins. Since the Pyd isoform expressed in nephrocytes, Pyd-P, has poor solubility in vitro, we resorted to use a more soluble N-terminal deletion of the protein, Pyd-PΔCC [36]. In salivary glands, both Pyd-PΔCC and Pyd-P colocalize with Scramb1-A-V5 in the plasma membrane (Fig. S4, B and C). Notably, Pyd-PΔCC was co-immunoprecipitated with Scramb1-A-V5, indicating an in vivo interaction between the two proteins (Fig. 4A). This interaction was further examined using a proximity labelling Bio-ID approach [37]. Pyd-P was fused to TurboID and expressed together with Scramb1-A-V5 in salivary glands. We found that Scramb1-A-V5 was biotinylated, indicating close proximity between the two proteins. In contrast, Scramb1-A-V5 was not biotinylated when coexpressed with TurboID-V5 as a control (Fig. 4B). These results support the hypothesis that Pyd helps recruit Scramb1-A to the SD pre-complexes. This is consistent with the observation that in nephrocytes of the null allele *pyd*^*ex147*^, Scramb1-A-V5 remains mostly cytoplasmic, with minimal overlap with Duf and Sns, which localize to cell-cell contact sites (Fig. 4C, arrows). In contrast, in *duf*^*sps1*^ nephrocytes, which lack SDs [10], Scramb1-A-V5 colocalizes with Pyd and Sns in cortical foci (Fig. S4 D).

**Fig. 5:**
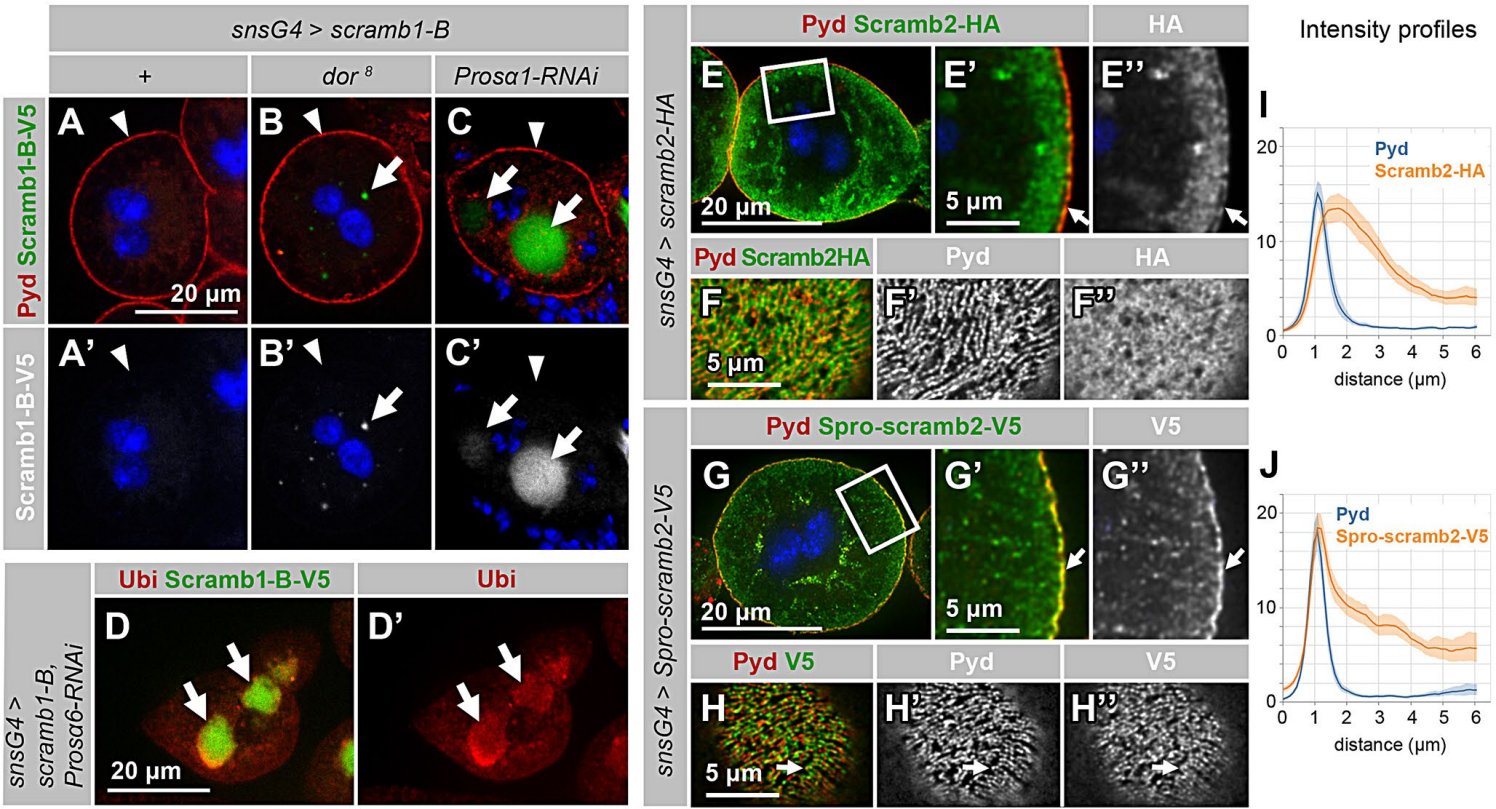
Scramb1-A proline-rich domain is required for protein stability and localization to SDs. **(A-C’)** Nephrocytes overexpressing the isoform Scramb1-B-V5 driven by *sns-GCN-Gal4* in an otherwise wild-type background (+), and in nephrocytes with compromised protein degradation via the lysosome pathway (*dor*^*8*^ mutants) or the proteasome pathway (*Prosα1* silencing), immunostained to reveal Scramb1-B-V5 (anti-V5 antibody) and Pyd in medial sections, as indicated. Scramb1-B-V5 is undetectable in A (+), but accumulates in cytoplasmic vesicles in *dor*^*8*^ (arrows in B-B’) and in aggresomes in Prosα1 depleted nephrocytes (arrows in C-C’). Scramb1-B-V5 does not accumulate in SDs (arrowheads). **(D-D’)** Silencing the proteasome subunit *Prosα6* in nephrocytes expressing *UAS-scramb1-B-V5* (*sns-GCN-Gal4*) results in the formation of aggresomes, identified by the accumulation of Ubiquitin (arrows), that also contain Scramb1-B-V5. Medial sections are shown. **(E-H’’)** Nephrocytes expressing Scramb2-HA or the chimera Spro-scramb2-V5 (*S*cramb1-A *pro*line-rich region fused to Scramb2), driven by *sns-GCN-Gal4*, stained with anti-Pyd and anti-HA or anti-V5, as indicated. E-E’’ and G-G’’ depict medial sections. The boxed regions in E and G are magnified in E’-E’’ and G’-G’’ respectively. Scramb2-HA accumulates at similar levels in the plasma membrane (arrows in E’-E’’) and the subcortical region, whereas Spro-scramb2-V5 accumulates at higher levels in the plasma membrane, colocalizing with Pyd (arrows in G’-G’’). The corresponding intensity profiles for Pyd and Scramb2-HA or Spro-scramb2-V5, expressed in arbitrary units, are shown in **I** and **J**, as indicated. The plasma membrane was registered at the 1 µm position. (F-F’’) Cortical section displaying partial colocalization between Pyd and Scramb2-HA, indicated by a Pearson’s colocalization coefficient of 0.335. (H-H’’) Cortical sections showing the distribution of Pyd and Spro-scramb2-V5’, colocalizing in a fingerprint-like pattern (arrows) with a Pearson’s colocalization coefficient of 0.516. Nuclei are labeled with DAPI (blue). A-C’ shown at the same magnification

As mentioned above, isoform Scramb1-A, but not Scramb1-B, contains an N-terminal region of 183 amino acids that is rich in proline residues and that could potentially mediate its targeting to the SDs by interacting with Pyd. To investigate the significance of this region, we generated a *UAS-scramb1-B-V5* transgene. Surprisingly, its expression in nephrocytes resulted in no detectable protein (Fig. 5, A and A’). However, it can readily be detected in wing imaginal discs after induction using the *hh-Gal4* driver, even though at lower levels than Scramb1-A-V5 (Fig. S4, E and F). These results suggest that the *UAS-scramb1-B-V5* transgene is translated, but the isoform Scramb1-B, lacking the proline-rich region, is unstable and degraded, particularly in nephrocytes. To test this possibility, we blocked both, the lysosomal and proteasome degradation pathways. Inhibiting lysosomal degradation by depleting the protein Vps18, which is involved in trafficking cargo to the late endosomes (*dor*^*8*^ mutant), led to the accumulation of Scramb1-B-V5 in cytoplasmic vesicles (Fig. 5, B and B’, arrows). Silencing several proteasome subunits by RNA interference, namely Prosα1, Prosα6 and Prosβ3 [38], resulted in a strong accumulation of Scramb1-B-V5 in cytoplasmic aggresomes, identified by anti-ubiquitin immunoreactivity (Fig. 5, C-D’, arrows; and Fig. S4 G). Thus, Scramb1-B is degraded in nephrocytes by the lysosomal and proteasomal pathways. Significantly, even after blocking its degradation, we did not observe colocalization with SD markers (Fig. 5, A-C’, arrowheads). This suggests that the proline-rich region of Scramb1-A is crucial for its stability and accumulation in SDs.

Next, we tested whether the proline-rich region of Scramb1-A could confer targeting to SDs when fused to *Drosophila* Scramb2 in the chimera *UAS-spro-scramb2-V5*. Upon expression of *UAS-scramb2-HA* in nephrocytes, the protein accumulated to comparable levels in the subcortical region, where the labyrinthine channels are located, and in the plasma membrane (Fig. 5, E-E’’, arrows points to the plasma membrane; and I). In cortical sections, Scramb2-HA does not describe the fingerprint-like pattern characteristic of SDs (Fig. 5, F-F’’). In contrast, the chimeric protein displayed an enhanced signal in the plasma membrane, where it colocalized with Pyd (Fig. 5, G-G’’, arrows; and J). In cortical sections, Spro-scramb2-V5 colocalized with Pyd in a fingerprint-like pattern (Fig. 5, H-H’’, arrows). These findings provide additional support to the notion that the proline-rich region of Scramb1 promotes SD targeting. However, it is noteworthy that the expression of this chimeric protein in *scramb1*^*43*^ nephrocytes failed to rescue its phenotype (Fig. S4, H and I).

### The putative Ca^2+^ binding domain in Scramb1-A is required for SD assembly but not for its localization to SDs

**Fig. 6:**
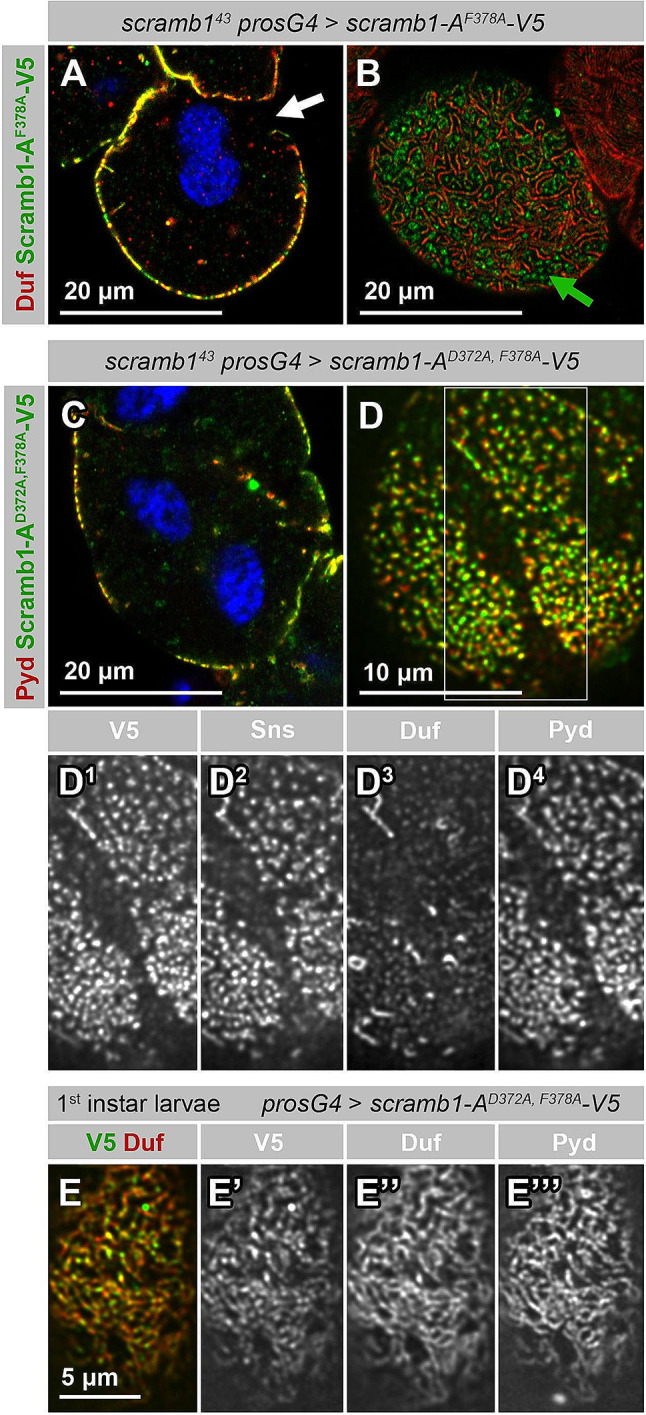
Requirement of the putative Ca^2+^-binding region of Scramb1-A. **(A-B)** Immunostaining of *scramb1*^*43*^ nephrocytes partially rescued by the expression of Scramb1-A^F378A^-V5, containing one residue substitution within its putative Ca^2+^ binding region, driven by *pros-Gal4* and shown at medial (A) and cortical (B) planes. The nephrocyte surface is partially covered by short SD strands identified by Duf accumulation, that coexist with foci containing Scramb1-A^F378A^-V5 and low levels of Duf (green arrow). White arrow in A points to a region devoid of SDs. **(C-D**^**4**^**)** Immunostaining of *scramb1*^*43*^ nephrocytes expressing Scramb1-A^D372A, F378A^-V5, a variant containing two residue substitutions within its putative Ca^2+^ binding region, driven by *pros-Gal4*, shown at a medial (C) and a cortical view at a higher magnification (D). The highlighted region in D is also shown as single channels (D^1^-D^4^), as indicated. Very few SDs are formed. Pyd, Sns and Scramb1-A^D372A, F378A^-V5 (anti-V5 antibody) accumulate in abundant cortical foci that contain low levels of Duf. **(E-E’’’)** Cortical view of a first instar larval nephrocyte expressing Scramb1-A^D372A, F378A^-V5 (*pros-Gal4*), immunostained as indicated. Similarly to Scramb1-A, this Ca^2+^-insensitive variant accumulates in SDs, identified by Duf and Pyd co-expression. (A, C) Nuclei were labeled with DAPI (blue). D-D^4^ shown at the same magnification

We have shown that Scramb1-A localization to pre-SD complexes is required for SD assembly and, in particular, for Duf recruitment. However, the molecular role that Scramb1-A plays within the SD complex remains unclear. Homologous proteins, such as human PLSCR1, have been shown to have phospholipid scrambling activity in vitro and to participate in membrane-driven processes in several cellular contexts [31, 32, 39, 59]. These activities are regulated by Ca^2+^ binding to a short 12-residue sequence with homology to the loop region of EF hand domains [40], a region conserved in Scramb1 (Fig. S1 F; and Fig. S5 A). To examine the role of this putative Ca^2+^ binding domain, we engineered three transgenes, each containing a single mutation in conserved residues previously shown to be required for PLSCR1 activity [40]. Expression of Scramb1-A^D372A^-V5 or Scramb1-A^F374A^-V5 in *scramb1*^*43*^ nephrocytes rescued SD formation at a similar level than the wild-type protein, as seen by the distribution of Duf in a characteristic fingerprint pattern in the cortical region and the absence of nephrocytes agglutination (Fig. S5, B-C’). The expression of Scramb1-A^F378A^-V5 resulted in a partial rescue of SD formation, with nephrocytes showing reduced SD density and cortical regions devoid of SDs (Fig. 6, A and B, white arrow). Interestingly, this mutant protein also accumulated in cortical foci that lacked or displayed low levels of Duf (Fig. 6B, green arrow).

Lastly, expression of Scramb1-A^D372A, F378A^-V5, a variant with mutations in two of the conserved residues, failed to rescue SD formation. Nephrocytes were agglutinated and lacked or displayed only a few SD strands. Moreover, a significant portion of the nephrocyte cortex was covered by foci that accumulated high levels of Scramb1-A^D372A, F378A^-V5, Pyd and Sns, but showed substantially reduced levels of Duf (Fig. 6, C-D^4^). These foci are similar to the ones observed in *scramb1*^*43*^ mutant nephrocytes (compare with Fig. 3A). Together, these data indicate that Scramb1 putative Ca^2+^ binding region is required to promote SD formation but not for Scramb1 colocalization with SD components. In fact, when Scramb1-A^D372A, F378A^-V5 is expressed in a wild-type background, it partially accumulates in SDs (Fig. 6E-E’’’).

### Palmitoylation of Scramb1 is essential to promote SD formation

To explore the cellular processes dependent on Scramb1 activity during SD formation, we undertook a proteomic approach to identify Scramb1 interactors. We generated a transgene expressing Scramb1-A fused with protein-A, enabling efficient purification of protein complexes by affinity chromatography. This fusion protein retains functionality, as it rescues SD formation in *scramb1*^*43*^ nephrocytes (Fig. S5, D and E). We expressed Scramb1-A-protA in the larval fat body, a large organ of mesodermal origin, and purified it from larval extracts, identifying 72 co-purifying proteins not present in a control experiment using an empty matrix (Supplementary Table 1). 23 putative interactors were mitochondrial proteins, likely reflecting a mitochondrial role for *scramb1* in the fat body, where it is also expressed. These interactors are unrelated to SD formation and therefore, were not further examined. We focused our attention on the interactors located at the plasma membrane and within the endo-lysosomal vesicular system, as they could potentially function alongside Scramb1 in promoting the assembly of SDs.

**Fig. 7:**
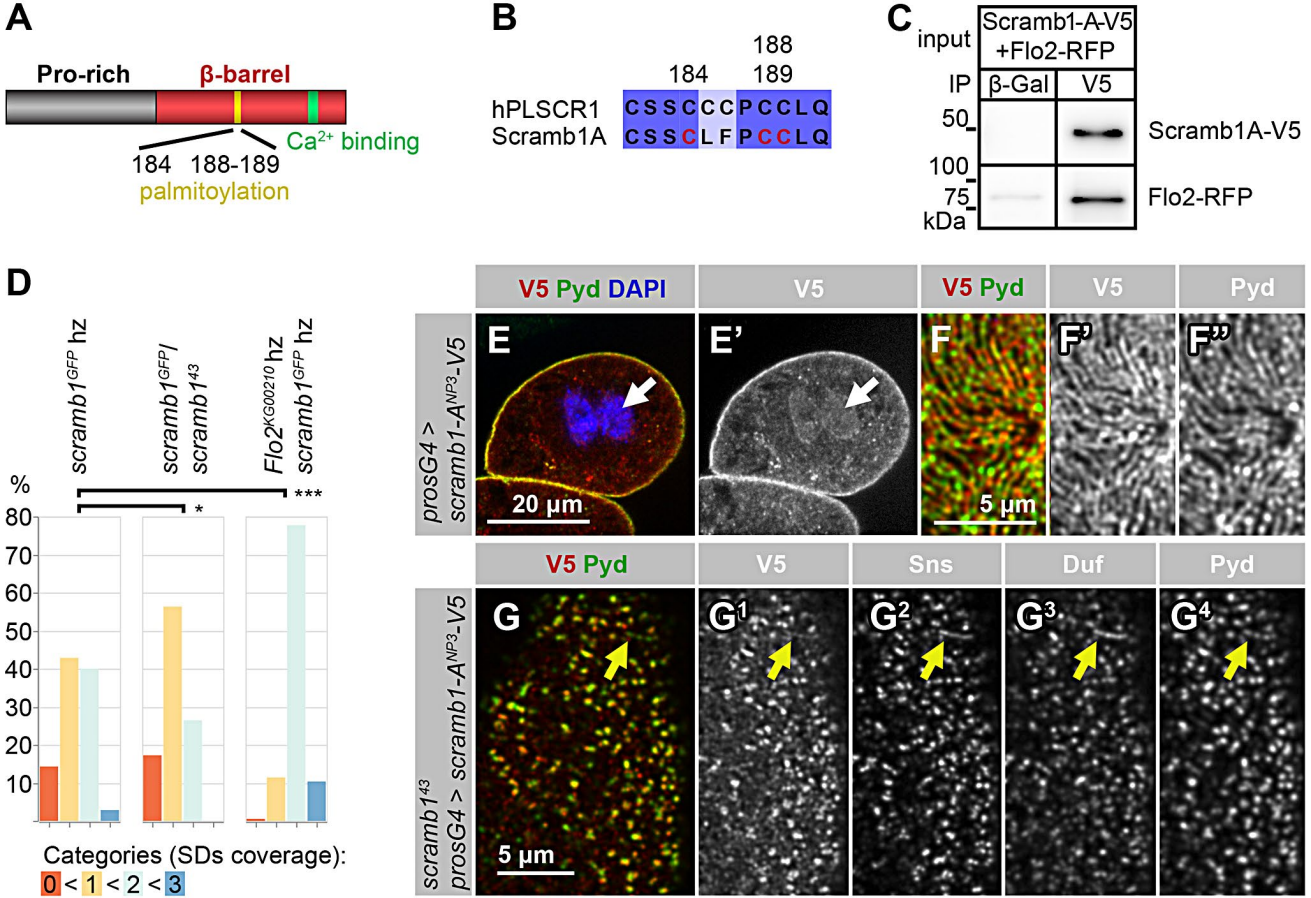
Requirement of Scramb1-A palmitoylation sites. (**A-B**) Scheme of Scramb1-A domain composition, highlighting a cluster of conserved cysteine residues (yellow) matching the human PLSCR1 palmitoylation site and sequence alignment of the region (**B**). The construct UAS-Scramb1-ANP3-V5A contains mutations in residues 184, 188 and 189, in red. A conserved putative Ca2+- binding site (green) is also indicated. (**C**) Co-IP from salivary glands coexpressing Scramb1A-V5 and Flo2-RFP. The extract was incubated with a magnetic matrix coupled to anti-V5 or to anti-?-galactosidase as a control, and the eluates analyzed by western blot to detect Scramb1-A-V5 (anti-V5 antibody) and Flo2-RFP (anti-RFP), as indicated. Flo2-RFP was co-immunoprecipitated with Scramb1-V5. (**D**) Genetic interaction between *scramb1* and *Flo2*. Three genotypes were quantitated: *scramb1*/*scramb1* (n = 105 cells), *scramb1*/*scramb1* (n = 87 cells) and a double mutant combination *flo2*/ Y; *scramb1*/*scramb1* (n = 193 cells). Nephrocytes were immunostained for Duf and Pyd and classified into four categories, from no SD strands observed (0) to SDs covering the entire nephrocyte surface (3). Examples are shown in Fig. S7. A mutation in *Flo2* normalizes the *scramb1* phenotype. Asterisks show statistical significance (* *P* < 0.05, *** *P* < 0.001). (**E-F’’**) Subcellular localization of non-palmitoylable Scramb1-ANP3-V5 driven by *pros-Gal4*. Immunostainings with anti-V5 and anti-Pyd are shown in medial planes (**E-E’**) and in cortical planes at higher magnification (**F-F’**). Scramb1-ANP3-V5 colocalizes with Pyd in SDs (**F-F’’**) and also accumulates in nuclei, colocalizing with DAPI in blue (arrows). (G-G) *scramb1* nephrocytes expressing UAS-scramb1-ANP3-V5 (*pros-Gal4*), immunostained to show the cortical distribution of Scramb1-ANP3-V5 (anti-V5 antibody), Sns, Duf and Pyd in foci and occasional short SD strands (yellow arrows). E-E’, F-F’’ and G-G shown at the same magnification

Among the potential Scramb1-A interactors at the plasma membrane was Flotillin2 (Flo2), a conserved protein found in cholesterol-rich membrane microdomains [41]. We validated this interaction through co-immunoprecipitation assays in salivary glands coexpressing *UAS-scramb1-A-V5* and *UAS-Flo2-RFP* (Fig. 7C).

Supporting the interaction of Flo2 with Scramb1 in nephrocytes, overexpressed Flo2-RFP, in addition to a cytoplasmic distribution, colocalized with the SD marker Duf in the plasma membrane (Fig. S6, A and A’, arrows). To assess the functional relevance of the interaction between Scramb1 and Flo2, we first examined Scramb1-A-V5 subcellular localization in the strong *Flo2*^*KG00210*^ allele, which lacks detectable levels of Flo2 and Flo1 proteins [42]. Scramb1-A-V5 distribution did not change compared to the control, indicating that the interaction with Flo2 is not essential for Scramb1A localization to SDs (Fig. S6, D and D’, compare to control C and C’). Moreover, in agreement with published data, SDs did not display gross alterations in *Flo2* mutants (Fig. S6, D and D’; and [22]). In contrast, after silencing *scramb1*, we observed decreased Flo2-RFP accumulation in the nephrocyte cortex, suggesting that the interaction between Scramb1-A and Flo2 promotes Flo2 accumulation in the plasma membrane harboring SDs (Fig. S6, B and B’, arrows).

To unveil a possible functional link between the two genes, we examined *Flo2* phenotype in SDs in a sensitized genetic background. To this end, we combined the strong *Flo2*^*KG00210*^ allele with *scramb1*^*MI01181 − GFSTF.0*^ (named *scramb1*^*GFP*^ for simplicity). In homozygosis, this *scramb1* hypomorphic allele is characterized by a reduced density of SDs or their absence in some nephrocytes, a phenotype that is sensitive to dosage since it increases when combined with the null allele *scramb1*^*43*^ (Fig. 7D and Fig. S7). When combined with *Flo2*^*KG00210*^ hemizygosity, the *scramb1*^*GFP*^ homozygous phenotype showed a tendency to normalize, pointing to opposing functions of the two genes during SD formation (Fig. 7D).

Flotillins are highly enriched in lipid raft membrane microdomains and serve as a marker for lipid rafts [41]. Thus, the physical interaction between Flo2 and Scramb1-A suggests that Scramb1-A is also present in lipid rafts. In this direction, human PLSCR1 partitioning into lipid rafts was suggested to be promoted by multiple palmitoyl adducts covalently bound to a cysteine cluster in PLSCR1 [28, 43, 44]. Interestingly, *Drosophila* Scramb1, which is also palmitoylated [45], contains a partially conserved cysteine-rich region that fits a consensus sequence for S-palmitoylation (Fig. 7, A and B). To examine the contribution of this region to Scramb1-A subcellular localization and function, we introduced mutations in three Cys residues in the region generating the transgene *UAS-scramb1-A*^*3NP*^*-V5* (Fig. 7A). When expressed in otherwise wild-type nephrocytes, Scramb1-A^3NP^-V5, besides localizing to the cortex in SDs (Fig. 7F-F’’), also accumulated in nuclei (Fig. 7, E and E’, arrows; compare with Fig. 1C), similarly to the non-palmitoylable form of hPLSCR [46]. Nevertheless, the lack of palmitoylation does not disrupt targeting to SDs, indicating that the non-palmitoylated protein can still interact with SD components (Fig. 7, F-F’’).

Next, we examined whether the non-palmitoylable Scramb1-A^3NP^-V5 protein could rescue SD formation when expressed in *scramb1*^*43*^ nephrocytes. This was not the case. In most nephrocytes, Pyd and Sns were distributed in the cortex in circular foci that also contained Duf at reduced levels, coexisting with a few thin strands corresponding to SDs. This phenotype is similar to that of the *scramb1*^*43*^ mutant, except for the foci also containing Duf (Fig. 7, G-G^4^, yellow arrows point to a short SD strand). Note that non-palmitoylable Scramb1-A^NP3^-V5 was still recruited to these foci, indicating that its failure to rescue the phenotype was not caused by mislocalization.

Together, our data shows that palmitoylation of Scramb1 is required to promote SD formation. Palmitoylation likely facilitates Scramb1 interaction with lipid raft micro-domains of the plasma membrane containing Flo2, although it is dispensable for Scramb1 localization with SD components.

### A potential role of Scramb1 in membrane remodeling in nephrocytes

**Fig. 8:**
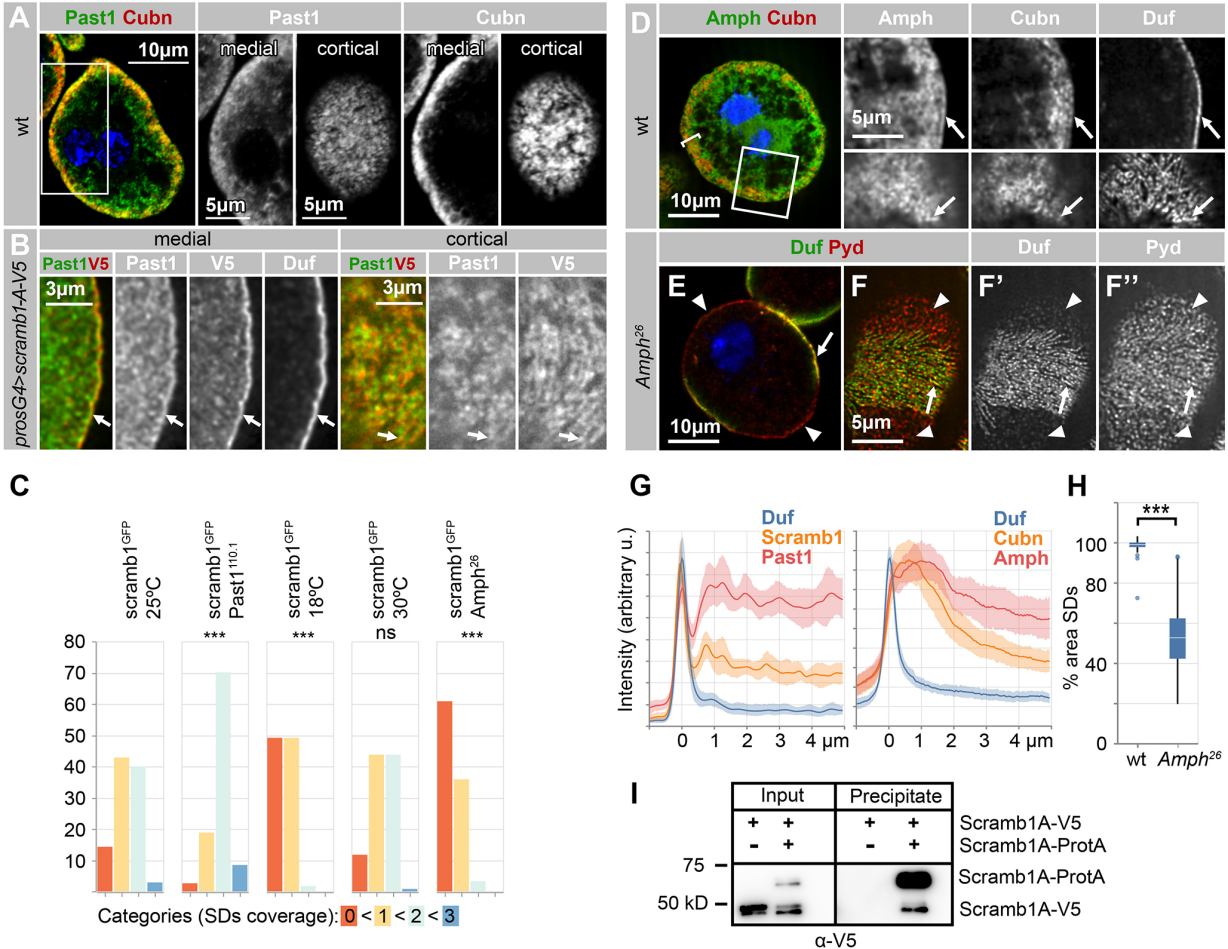
Scramb1 interaction with membrane-remodeling proteins. **(A)** Wild-type nephrocyte displaying the distribution of Past1 and Cubn, as indicated. Higher magnification, single-channel data are presented at medial sections, corresponding to the boxed region, as well as at cortical levels. Past1 expression is higher in the labyrinthine channels regions, marked by Cubn expression. **(B)** Immunostaining of a nephrocyte expressing *UAS-Scramb1-A-V5* driven by *pros-Gal4*, depicting the distribution of Past1, Scramb1-A-V5 (anti-V5 antibody), and Duf in high magnification images. At medial sections, Past1 is detected subcortically and at the plasma membrane, alongside Duf and Scramb1-A-V5 (arrows). In cortical sections, Past1 partially overlaps with Scramb1-A-V5, with a Pearson’s colocalization coefficient of 0.763. Arrows points to one region of overlap, as reference. **(C)** Quantitation of the nephrocyte phenotypes in the following genotypes and conditions: *scramb1*^*GFP*^/ *scramb1*^*GFP*^ at 25 °C (*n* = 105 cells), 18 °C (*n* = 118 cells) and 30 °C (*n* = 128 cells) and in the double mutant genotypes: *scramb1*^*GFP*^, *Past1*^*110.1*^/*scramb1*^*GFP*^, *Past1*^*110.1*^ (*n* = 154 cells) and *Amph*^*26*^/*Amph*^*26*^; *scramb1*^*GFP*^/*scramb1*^*GFP*^ (*n* = 92 cells) at 25 °C. Nephrocytes were classified into four categories ranging from the absence of SDs (category 0) to SDs covering the entire nephrocyte surface (category 3, examples shown in Fig. S7). The statistical significance of the observed differences compared to *scramb1*^*GFP*^ at 25 °C is indicated (ns: non-significant, *** *P* < 0.001). **(D)** Distribution of Amph, Cubn and Duf in wild-type nephrocytes, as indicated. Amph exhibits a cytoplasmic distribution with a preferential accumulation in the labyrinthine channels region, identified by Cubn expression (bracket). Amph is also detected in the plasma membrane, displaying partial colocalization with Duf (arrows), as depicted in high-magnification single-channel images of medial section (upper panels) and cortical sections (lower panels. Pearson’s colocalization coefficient for Amph-Duf: 0.577). **(E-F’’)** Medial (E) and cortical (F’-F’’) sections of *Amph*^*26*^ mutant nephrocytes illustrating the distribution of SDs (anti-Duf and anti-Pyd), which decorate only a fraction of the nephrocyte surface (arrows), while the remaining surface exhibits low Duf levels and Pyd accumulation in foci and short rods (arrowheads). **(G)** Intensity profiles across medial sections of the nephrocytes shown in B (Duf, Scramb1-A-V5 and Past1) and in wild-type nephrocytes (Duf, Cubn and Amph). The plasma membrane is registered to the 0 μm position. **(H)** Quantitative analysis of the *Amph*^*26*^ phenotype, depicting the fraction of cell surface containing SDs for 19 wild-type and 19 *Amph*^*26*^ nephrocytes. **(I)** Scramb1-A oligomerization. Lysates from salivary glands coexpressing Scramb1-A-ProtA and Scramb1-A-V5 or only Scramb1-A-V5 (control), were incubated with a matrix conjugated to rabbit IgGs to precipitate Scramb1-A-ProtA. Lysates (input) and eluates (precipitate) were analyzed by western blot with anti-V5, which detects both Scramb1-A-V5 and Scramb1-A-ProtA. Scramb1-A-V5 co-precipitates with Scramb1-A-ProtA, indicating a capacity to oligomerize. A, D and E, nuclei are labeled with DAPI (blue)

Our proteomics analysis identified Past1, the sole *Drosophila* member of the C-terminal EHD protein family, as a Scramb1-A interactor. EHD proteins are large, membrane-binding ATPases that share structural similarities to dynamin, have the capacity to induce the formation of membrane tubules in vitro and play key functions in ligand recycling [47]. Thus, Past1 could potentially participate in membrane remodeling processes during SD formation, alongside Scramb1. To examine this possibility, we analyze Past1 distribution and activity in nephrocytes. Past1 accumulates at higher levels in the nephrocyte subcortical region occupied by the labyrinthine channels, marked by the expression of the endocytic receptor Cubn (Fig. 8A). Additionally, Past1 is detected in the plasma membrane, where it partially colocalizes with Scramb1-A-V5 and Duf (Fig. 8, B, arrows, G; and [48]). *Past1* mutant nephrocytes display attenuated endocytosis even though the cortical distribution of SDs is not grossly affected ([48]; and Fig. S6, E and F). To examine whether *Past1* functionally interacts with *scramb1*, we tested for a genetic interaction between the null allele *Past1*^*110.1*^ and the hypomorphic allele *scramb1*^*GFP*^. When these alleles were combined, the area of the nephrocytes covered by SD strands increased compared to the homozygous *scramb1*^*GFP*^ allele, indicating a negative functional interaction between these genes (Fig. 8C, and Fig. S7). Thus, Past1 activity opposes the SD-promoting function of Scramb1, opening the possibility that the physical interaction between the two proteins is required for their mutual inhibition.

Scramb1 interaction with Past1 led us to explore the possible involvement of other membrane remodeling proteins in Scramb1-mediated SD formation, focusing on the BAR-domain protein Amphiphysin. In recycling endosomes, C-terminal EHD proteins form complexes with Amphiphysin and the F-BAR domain protein Syndapin [49]. In *Drosophila*, Past1, together with Amphiphysin (Amph) and Syndapin, control the formation of complex postsynaptic membrane elaborations found at the neuromuscular junctions [50]. We observed that Amph, similarly to Past1, exhibits elevated expression within the subcortical labyrinthine channels region, identified by Cubn expression (Fig. 8, D, bracket; and G), and it is also detected at the plasma membrane, where it partially colocalizes with Duf (Fig. 8, D, arrows; and G).

Examination of *Amph*^*26*^ mutant nephrocytes revealed that certain areas in the cortex exhibited SD strings harboring both Duf and Pyd (Fig. 8, E-F’’, arrows), contrasting with other regions characterized by diminished Duf levels and Pyd accumulating in foci and short rods (Fig. 8, E-F’’, arrowheads. Quantitated in H). This phenotype suggests that Amph plays a role in SD formation or maintenance, particularly in the stabilization of Duf within these complexes. Interestingly, in the double mutant *Amph*^*26*^; *scramb1*^*GFP*^, the loss of SDs was more pronounced than in either single mutant, supporting a functional link between these two genes (Fig. 8C; and Fig. S7).

Altogether, our results suggest that Scramb1 assists in the formation of multiprotein complexes containing SD core components as well as general factors such as flotillins and Past1. Building such complexes is typically favored by the homo- and hetero- oligomerization of the network components. Thus, we examined whether Scramb1-A can multimerize in vivo. To this end, we coexpressed two Scramb1-A variants, one tagged with V5 and the other with Protein A, in salivary glands. After pulling-down Scramb1-A-Protein A from the lysate using a matrix coupled to IgGs, the precipitate also contained Scramb1-A-V5, indicating formation of Scramb1-A homo-oligomers (Fig. 8I).

The ability of Scramb1-A to oligomerize, coupled with its physical interaction with Flo2 and Past1, and its genetic interaction with *Amph*, favors a model where Scramb1 helps assemble a protein network in lipid raft microdomains of the plasma membrane with the capacity to remodel membranes. In this direction, we observed a significant enhancement of the phenotype of the *scramb1*^*GFP*^ hypomorphic allele when perturbing membrane rigidity by culturing the flies at 18 °C compared to the control temperature of 25 °C [51], whereas no modification of the phenotype was observed by increasing the temperature to 30 °C (Fig. 8C; and Fig. S7). Thus, Scramb1 phenotype in SD formation is sensitive to basic properties of the membrane.

## Discussion

The slit diaphragm junction is a core component of the filtration apparatus in both kidney glomeruli and *Drosophila* nephrocytes. Currently, more than 20 proteins are known to localize at the SD complex, either as main structural components or dynamically associated with it, including transmembrane adhesion proteins, ion channels, cytoplasmic adaptors, scaffolding proteins or signaling molecules [52]. However, our understanding of the mechanisms and intermediary stages involved in the assembly of this protein complex is still limited.

Here, through the identification and characterization of Scramb1 as a novel component of the SDs, we unveil a critical requirement of this protein during the process of SD assembly. In the absence of Scramb1, certain aspects of SD formation still take place. Notably, Sns is targeted to the plasma membrane at the nephrocyte surface in contact with the basement membrane and hemolymph, forming circular foci alongside Pyd and the activated form of the kinase Src64B. Importantly, Duf is absent from these foci, implying that Scramb1 is required to recruit Duf to SDs during their assembly. This interaction is likely indirect, as suggested by the inability of Duf to recruit Scramb1 in S2 cells or in *pyd* mutant nephrocytes, where SDs do not form. Given the known association between Duf and Pyd [10], Scramb1 might be promoting Duf recruitment by modulating the strength of its interaction with Pyd. Interestingly, similar cortical foci containing Pyd and Sns are also observed in *duf* mutants ([36, 53]; and Fig. S4 D), suggesting that defective Duf recruitment contributes to the lack of SDs observed in *scramb1* mutants. Furthermore, our observation that a non-palmitoylable Scramb1 variant fails to promote SD formation, despite the presence of Duf within foci containing other SD components (Fig. 7, G-G^4^), indicates that Scramb1 likely fulfills additional functions independent of Duf recruitment.

To gain insight into Scramb1 role in SD formation, we conducted structure-function studies. Most PLSCRs consist of an unstructured, poorly conserved, N-terminal proline-rich domain of variable length, followed by a globular, 12-stranded β-barrel domain, with a C-terminal hydrophobic α-helix suggested to be buried within the β-barrel central cavity [30, 54, 55]. We found that the proline-rich region of *Drosophila* Scramb1-A probably mediates its interaction with Pyd. This is in line with the described function of this region in facilitating protein-protein interactions and oligomerization in other PLSCRs [56–58]. This interaction with Pyd is largely independent of Scramb1-A activity, since our results indicate that Scramb1 proteins containing mutations in the putative Ca^2+^-binding or palmitoylation sites still colocalizes with Pyd, despite being unable to mediate SD formation.

To examine the role of the globular β-barrel domain, we mutated a putative Ca^2+^ binding site in this domain known to be essential for the phospholipid scramblase activity of PLSCR1 both in vitro [40] and in vivo [31, 59]. The Ca^2+^-insensitive Scramb1 protein failed to promote SD formation, indicating a requirement of Ca^2+^ binding for Scramb1 activity. Remarkably, Ca^2+^ signaling is critical for podocyte foot process formation and has also been implicated in SD formation and repair in both vertebrates [60], and in *Drosophila* [61]. Thus, our findings point towards an exciting possibility: that Ca^2+^ signaling may control the assembly of SDs by impacting the activity of Scramb1 and affecting Duf recruitment, a mechanism that could involve conformational changes in Scramb1 and that should be further explored.

Finally, our observations indicating that a chimeric protein containing the proline-rich domain from Scramb1 and the globular β-barrel domain from Scramb2 do not promote SD formation despite localizing at the membrane, suggest that the β-barrel domain is also involved in stablishing essential interactions with other proteins.

### Scramb1 localization within lipid raft microdomains

We hypothesize that Scramb1 associates with the plasma membrane within lipid raft microdomains. This suggestion is grounded in several key observations. First, *Drosophila* Scramb1 undergoes palmitoylation, a post-translational modification known to facilitate localization within lipid rafts [45, 62]. Additionally, we observed a physical interaction between Scramb1 and Flo2, a protein highly enriched in lipid raft microdomains [42], as well as with Hip14, a palmitoyl transferase potentially involved in Scramb1 palmitoylation (Table S1). Importantly, we found that mutations in conserved cysteine residues within the putative Scramb1 palmitoylation site disrupt its ability to mediate SD formation, even though it still colocalizes with Pyd, Sns and Duf. This observation underscores the necessity of lipid raft localization for Scramb1 functionality and the assembly of SDs. However, it is worth noting that the interaction with Flo2 is not an absolute requirement, as evidenced by our findings and others’ observations of SD formation in *Flo2* mutants [22].

Currently, it is well established that the SD complexes of human podocytes localize within lipid raft microdomains, and this localization is essential for their proper function [14, 15, 18, 19, 21, 22]. The association of podocyte SDs with lipid raft microdomains depends, at least in part, on Podocin, a stomatin family protein that associates with the inner leaflet of the plasma membrane and in particular, is targeted to lipid rafts by its palmitoylated Prohibitin domain, which can directly interact with Flo2 [17, 20, 21]. Podocin interactions with nephrin, NEPH1 and CD2AP, along with its multimerization capacity, contribute to the stabilization of these proteins within lipid raft domains [19, 21, 63]. Similarly, in *Drosophila*, we propose that Scramb1 is targeted to lipid rafts through its palmitate adducts, where it promotes the stabilization of other SD components. This process is facilitated by Scramb1 ability to interact with Pyd and Flo2 and its multimerization capacity (Model in Fig. S8).

SD formation in mammals might require activities similar to those fulfilled by Scramb1 in *Drosophila*. Although till date no known functions have been ascribed to PLSCR proteins during SD formation in mammals, it is worth mentioning that transcriptomic data indicate that PLSCR2, a mostly uncharacterized protein, is enriched in podocytes in mice [64], and that PLSCR1 is one of the genes with most decreased expression in glomerular samples from patients with diabetic nephropathy [65], making these genes good candidates for further exploration. Alternatively, as suggested by the functional similarities previously mentioned between Scramb1 and Podocin, proteins with no sequence homology to Scramb1 might play equivalent cellular functions in mammals as those of Scramb1 in *Drosophila*.

### Scramb1 role in membrane remodeling during SD formation

It is important to note that despite the homology between Scramb1 and PLSCR1, a possible phospholipid scrambling activity for the *Drosophila* protein remains an open question. In particular, results by Acharya et al. [33] challenged this assumption, as *scramb1* knock-down or overexpression in S2 cells did not change phosphatidylserine exposure. Beyond phospholipid scrambling, PLSCR proteins were shown to participate in cellular processes that result in profound membrane remodeling events. These include PLSCR1 involvement in mediating the translocation of Akt and PLSCR1 itself from the cytosolic to the extracellular side of the membrane during herpes simplex virus cellular entry, an activity associated to phospholipid scrambling [32], its anti-fusogenic activity during SARS-CoV-2 infection, which does not involve phospholipid scrambling [59] and PLSCR1 regulation of compensatory endocytosis in neuroendocrine cells, associated to scrambling [31]. Similarly, we posit that Scramb1 participates in membrane remodeling processes occurring during SD assembly, which may result in incipient membrane invagination required to facilitate the interaction between the extracellular domains of Duf and Sns and the formation of the SD molecular filter (Model in Fig. S8). This is supported by our findings, which indicate physical and/or genetic interactions between Scramb1 and the membrane-remodeling proteins Past1 and Amph. Additionally, the SD-promoting activity of Scramb1 is strongly reduced at low temperatures, a conditions that affect the biophysical properties of membranes. In this context, a possible Ca^2+^-dependent phospholipid scramblase activity might facilitate the local remodeling of membranes by alleviating tensions in phospholipid packing generated by the bending of membranes. However, to fully elucidate the precise roles of membrane remodeling proteins during SD assembly and the relevance of a phospholipid scrambling activity in Scramb1, further investigations will be required.

Having shown the critical role played by Scramb1 during SD assembly, it was puzzling to observe the presence of scarce SDs in *scramb1* null mutant garland nephrocytes. These remnant SDs tend to be located close to cell-cell contacts between agglutinated nephrocytes and can also be observed in early larvae. We speculate that these extant SDs might derive from the first SDs formed during embryogenesis, which were recently shown to require specialized processes [23]. In particular, these early SDs assemble in PI(4,5)P2 enriched membrane domains and associate to membrane remodeling events occurring during the formation of a cytokinetic ring formed during an acytokinetic cell division [23]. These processes might render Scramb1 function redundant during the formation of these initial SDs in embryonic garland nephrocytes.

## Materials and methods

### Drosophila genetics

The novel allele *scramb1*^*43*^ was generated by the imprecise excision of the P element EY07744 [66, 67]. Two additional alleles displayed identical phenotypes. The novel *scramb2*^*V3*^ allele was generated by CRISPR-Cas9 genome editing [68], targeting *scramb2* first coding exon. The double mutant chromosome *scramb2*^*V6*^, *scramb1*^*43*^ was similarly generated on a *scramb1*^*43*^ background. The *scramb2*^*V3*^ allele harbors a deletion of one nucleotide, leading to a frameshift mutation after residue Asp-17, while in *scramb2*^*V6*^, a microdeletion of 10 nucleotides causes a frameshift mutation after residue Gln-16. The recovered alleles were characterized by PCR amplification of the relevant genomic regions followed by sequencing analysis. Other alleles and stocks used in this work are: wild-type Oregon R; *scramb1*^*MI01181 − GFSTF.0*^ (BDSC #60,164); *dor*^*8*^ (BDSC, #28); *Df(3R)pyd*^*ex147*^ [69]; *duf*^*sps1*^ [10]; *Past1*^*110.1*^ [48]; *Amph*^*26*^ [70] and *Flo2*^*KG00210*^ [42].

The following UAS lines were generated in this work by targeted transgenesis, utilizing the zh-22 A landing site [71]: *UAS-scramb1-A-V5*; *UAS-scramb1-B-V5*; *UAS-Pyd-turboID-V5*; *UAS-Spro-scramb2-V5*; *UAS-scramb1-A*^*D372A*^*-V5*; *UAS-scramb1-A*^*F374A*^*-V5*; *UAS-scramb1-A*^*D372A, F378A*^*-V5*; *UAS-scramb1-A*^*NP3*^ and *UAS-scramb1-A-Prot-A.* Other UAS lines and Gal4 drivers used are: *UAS-Scramb2-HA* (FlyORF, F002902); *UAS-Flo2-RFP* [72]; *UAS-pyd-P* and *UAS-pyd-P-ΔCC* [36]; *UAS-V5-TurboID* [37]; *UAS-RNAi-Prosα1*, *UAS-RNAi-Prosβ3* and *UAS-RNAi- Prosα6* (NIG-Fly #18495R-3, #11981R-3 and #4904R-2 respectively [38]); *UAS-RNAi-scramb1* (VDRC #107,024); *pros-Gal4* (a gift from Chris Q. Doe); *sns-GCN-Gal4* [11]; *AB1-Gal4* (BDSC, #1824); *Cg-Gal4* [73] and *hh-Gal4* [74].

To express *UAS-scramb1-A-V5* in nephrocytes for controlled periods of time in a *scramb1*^*43*^ background (experiments shown in Fig. 3 and Fig. S3) using the TARGET system [35], the following genotype was used: *UAS-scramb1-A-V5*, *tubP-GAL80*^*ts*^#10 /*tubP-GAL80*^*ts*^#20; *pros-Gal4*, *scramb1*^*43*^/*tubP-GAL80*^*ts*^#7, *scramb1*^*43*^. Three *tubP-GAL80*^*ts*^ transgenes (BDSC #7019, #7108 and #7018) were required to completely block *UAS-scramb1-A-V5* expression at the restrictive temperature of 18 °C. Larvae were switched to the permissive temperature of 29 °C for the required time intervals before dissection.

### DNA constructs

#### UAS-scramb1-A-V5

A DNA fragment coding for the complete *scramb1-A* ORF and containing engineered NotI and XbaI flanking restriction sites was generated by PCR amplification from cDNA GM13876 [75] using primers 1 and 2. A C-terminal V5 epitope tag was added through an intermediary cloning step into pAc5.1/V5-His B vector (invitrogen). A second PCR using primers 1 and 3 generated a fragment containing *scramb1-A* ORF fused to V5 and containing flanking NotI and Asp718 sites, which was transferred to pUASTattB [71].

#### UAS-scramb1-B-V5

A cDNA corresponding to isoform *scramb1-B* was obtained by reverse transcription from RNA of larval nephrocytes using oligonucleotide 4, as described in the “Reverse transcription” section. The reaction was amplified by PCR using oligonucleotides 5, which targets the 3’ end common to all isoforms, and 6, specific for isoform B 5’ end. The products were cloned and one cDNA corresponding to isoform B was selected by sequencing. A UAS-scramb1-B-V5 clone was generated following the same strategy as for UAS-scramb1-A-V5, except that oligonucleotide 7, specific for the 5’ region of isoform B, was used instead of oligonucleotide 2.

#### UAS-Pyd-turboID-V5

*pyd-P* clone MIP30509 [75] in the vector pOT2 was used as template. A BamHI site was engineered to replace the stop codon corresponding to isoform pyd-PP (GenBank: AFH06317.2) using oligonucleotide 10. A DNA fragment coding for V5-TurboID-NES including two stop codons and flanked by BamHI and XbaI sites was obtained by PCR amplification from pUAS-V5-TurboID-NES [37] using oligonucleotides 8 and 9. This and the previous DNA segments were fused in frame at the engineered BamHI site. Finally, an EcoRI-XbaI fragment comprising the *pyd-P* ORF fused to V5-TurboID-NES was cloned into pUASTattB through intermediary steps to account for an internal EcoRI site.

#### UAS-Spro-scramb2-V5

Using as templates UAS-scramb1-A-V5 and *scramb2* cDNA GH10494 [75], we generated a DNA fragment containing the proline-rich region of Scramb1-A, from M1 to G181, fused to the complete coding sequence of *scramb2*. The fragment, flanked by NotI and EcoRI sites and lacking the stop codon, was generated by overlapping PCR using oligonucleotides 13, 19, 20 and 21 and cloned into pGEM-T Easy. A second fragment containing a V5 epitope tag flanked by EcoRI at the 5’ end and a stop codon followed by a NotI site at the 3’ end, was obtained by PCR amplification using UAS-scramb1-A-V5 as template and the oligonucleotides 17 and 18. The two fragments were combined in the vector pGEM-T Easy and transferred to pUASTattB.

#### UAS-scramb1-A^D372A^-V5, UAS-scramb1-A^F374A^-V5**, **UAS-scramb1-A^D372A, F378A^-V5 and UAS-scramb1-A^NP3^

Directed mutagenesis of the Ca^2+^ and palmitoylation sites in Scramb1-A was performed using the Stratagene kit “Quick Change Mutagenesis”. To prepare the template, a NotI-Asp718 DNA fragment comprising Scramb1-A fused to V5 was excised from UAS-scramb1-A-V5 and cloned into pBS-SK. Directed mutagenesis was performed on this vector using the oligonucleotide pairs described below, sequenced to select the clones that only incorporated the desired mutations and transferred to pUASTattB. Oligonucleotides used: UAS-scramb1-A^D372A^-V5: 22 and 23; UAS-scramb1-A^F374A^-V5: 24 and 25; UAS-scramb1-A^D372A, F378A^-V5: 26 and 27; UAS-scramb1-A^NP3^: 30 and 31.

#### UAS-scramb1-A-Prot-A

A DNA fragment comprising a TEV protease cleavage site followed by two Protein A repeats and flanked by two engineered XbaI sites was obtain by PCR amplification using the oligonucleotides 32 and 33 and the vector pUAS-CTAP [76] as template. This fragment was cloned into pGEM-T Easy, released by XbaI digestion and used to replace the V5 tag in UAS-scramb1-A-V5.

The fidelity of all constructs obtained by PCR amplification was checked by sequencing.

### Oligonucleotides

The following oligonucleotides were used through this work:

1: GCGGCCGCGGGAAATCCCACGAAATGAAG

2: TCTAGAAGCATTCCGGGCCTATCGGTCTC

3: GGTACCGGTACGCGTAGAATC

4: CGTCGCAATCGCATTCGCAAT

5: CGTCGCCGCAGAGCGAAA

6: GTTCATCTCTGTGCTGCAG

7: GCGGCCGCACAGGAGGAGACTGGATG

8: CGGATCCATGGGCAAGCCCATCC

9: CTCTAGACTATTAGTCCAGGGTCAGGC

10: CGGATCCTGCAATGCATTCGTTACTTTGGC

11: CGAATTCGCCACCATGGAACAAAAGCTGA

12: GAGCGGCCGCTACCACACTC

13: CTGCGGCCGCGGGAAATCCCACGAAATG

14: ATCCATGGTCCTGCTGGTCCGCCCTGTG

15: ACCAGCAGGACCATGGATGCCAGCGCCACA

16: GCGAATTCCCACACTCCTGATTTTTGTTCCTGGC

17: GCGAATTCGGCCCGCGGTTCGAAGGT

18: GAGCGGCCGCTACGTAGAATCGAGACCGAG

19: ATCTGCATCATTCCTGCTGGTCCGCC

20: GACCAGCAGGAATGATGCAGATGAGCGAG

21: GCGAATTCCTGCTCGTAGTAAACCGCG

22: GAAATTTTCACGGATGCGGCCTTCTTCGGCATC

23: GATGCCGAAGAAGGCCGCATCCGTGAAAATTTC

24: CGGATGCGGACTTCGCCGGCATCAATTTCCC

25: GGGAAATTGATGCCGGCGAAGTCCGCATCCG

26: CACGGATGCGGCCTTCTTCGGCATCAATGCCCCACTGGACTTGG

27: CCAAGTCCAGTGGGGCATTGATGCCGAAGAAGGCCGCATCCGTG

28: TCCTGTCTGTTTCCCGCCGCTCTGCAGAGTATCGA

29: TCGATACTCTGCAGAGCGGCGGGAAACAGACAGGA

30: GCCTGCTCCTCCGCTCTGTTTCCCGCCGCTCTGCAGAGTATCGA

31: TCGATACTCTGCAGAGCGGCGGGAAACAGAGCGGAGGAGCAGGC

32: CCTCTAGATATTCCAACTACTGCTAGCG

33: GGTCTAGACTAGTTCGCGTCTACTTTCG

34: GGATGAGTATACCTACCGG

35: GATTCCAGCCAAATGCTCCG

### Reverse transcription

Total RNA was prepared from wild-type and *scramb1*^*43*^ garland nephrocytes partially dissected from 25 third instar larvae using the TRizol reagent (ThermoFisher). For each reverse transcriptase reaction, 1 µg of RNA and oligo 4 at 2 µM final concentration were combined in a volume of 13 µL. The reaction mixture was incubated at 65 °C for 10 min, the reverse transcriptase buffer, dNTPs, RNAse inhibitor and 20 units of AMV reverse transcriptase (Roche) added and the reaction incubated for 30 min at 55 °C followed by 5 min at 85 °C in a thermocycler. To detect the presence of *scramb1* transcripts in the wild-type and *scramb1*^*43*^ reactions, two different oligo pairs were designed targeting exons common to all isoforms (PCR B, oligonucleotides 5 and 34) or specific for isoform scramb1-A (PCR A, oligonucleotides 5 and 35) and used for PCR amplification from the reverse transcription reactions.

### Abundance of *scramb1* isoforms in nephrocytes

To estimate the relative abundace of scramb1-A and scramb1-B transcripts in nephrocytes, we examined RNA-seq data from FlyAtlas2 [34]. Reads from study PRJEB48667 (European Nucleotide Archive), sample SAMEA10748293 corresponding to dissected larval garland cells [34], were aligned to the *Drosophila* genome using RNA STAR software in the Galaxy platform. 97 paired reads were compatible with splicing between exons 1 or 2 and exon 4, corresponding to transcript scramb1-A, whereas 6 paired reads were compatible with splicing between exon 3 and exon 4, corresponding to transcript scramb1-B. Thus, in this sample, about 94% of *scramb1* transcripts code for isoform A. Of note, none of the reads supported the existence of transcript scramb1-C (FlyBase).

### Co-immunoprecipitation

Interaction between Pyd-P-ΔCC or Flo2 with Scramb1-A were analyzed by co-immunoprecipitation. 600 salivary glands from third instar larvae coexpressing *UAS-scramb1-A-V5* and either *UAS-pyd-P-ΔCC* or *UAS-Flo2-RFP* driven by *AB1-Gal4*, were dissected and lysates prepared by homogenization in 600 µL of lysis buffer (150 mM NaCl, 20 mM Tris-HCl pH 7.5, 0.5% Triton X-100, 1 mM DTT, 0.5 mM PMSF and Roche protease inhibitor cocktail). Non-soluble material was removed by centrifugation at 16,000 g for 30 min at 4 °C. Supernatants were divided in two equal volumes and incubated with 25 µL of Dynabeads Protein G (Invitrogen) slurry previously coupled to 3.5 µg of mouse anti-V5 (Invitrogen, #46–0705) or 3.5 µg of mouse anti-β-Galactosidase (Promega, #Z378A), as a control, during 30 min at room temperature. The matrix was washed 7 times with 200 µL of lysis buffer and eluted in 20 µL of Laemmli buffer at 95 °C. Elution fractions were analyzed by western blot to detect Scramb1-A-V5 (mouse anti-V5, 1% of the eluted fraction) and the co-immunoprecipitated Pyd-P-ΔCC (mouse anti-Pyd2, DSHB) or Flo2-RFP (mouse anti-RFP 6G6 Chromotek).

### Proximity labeling with TurboID

*UAS-scramb1-A-V5* was coexpressed with *UAS-Pyd-P-TurboID-V5* or with *UAS-TurboID-V5*, as a control, in the salivary glands of third instar larvae grown in culture media supplemented with 0.1 mM biotin, driven by *pros-Gal4*. 42 salivary glands were dissected and lysates prepared by homogenization in 200 µL of lysis buffer (150 mM NaCl, 30 mM Tris-HCl pH 7.8, 0.1% Triton X-100, Roche protease inhibitor cocktail and 0.5 mM PMSF). Insoluble material was removed by centrifugation at 16,000 g for 30 min at 4 °C and biotinylated proteins purified by incubation with 40 µL of Dynabeads MyOne Streptavidin C1 (Invitrogen) at 4 °C overnight. The matrix was washed 5 times in lysis buffer and proteins eluted by incubation in Laemmli buffer supplemented with 2 mM biotin at 95 °C during 5 min. Input (equivalent to 10% of the eluted fraction) and elution fractions were analyzed by western blot using anti-V5, which detects Scramb1-A-V5 as well as TurboID-V5 and Pyd-P-TurboID-V5.

### Affinity purification and identification of Scramb1-A interacting proteins

Third instar larvae expressing *UAS-scramb1-A-ProtA* in the fat body driven by *Cg-Gal4* were collected, washed and frozen in dry ice. Control *Cg-Gal4* larvae were similarly processed. 6 g of larvae for each genotype were grinded into a fine powder using a mortar and pestle previously cooled in dry ice. This powder was added to 50 mL of lysis buffer (50 mM Tris-HCl pH 7.5, 150 mM NaCl, 2 mM EDTA, 19 mM CHAPS, 0.5% Triton X-100, 5 mM DTT, Roche protease inhibitor cocktail, 1 mM PMDF), processed in a Potter homogenizer and incubated at 4 °C for 20 min. Insoluble material was removed by centrifugation at 15,500 g for 5 min to remove particulate material followed by two filtrations in 2.7 μm and 0.7 μm pore size filters fitted with a glass fiber pre-filter and a final centrifugation at 15,500 g for 15 min at 4 °C. The cleared lysates were incubated during 2 h at 4 °C with 200 µL of 50% slurry of Epoxi Dynabeads M-270 (Invitrogen) previously conjugated to rabbit IgGs following the protocol in [77]. The matrix was washed once in 50 ml and four additional times in 1 ml of lysis buffer excluding CHAPS and bound proteins eluted by a proteolytic cleavage with 150 units of TEV in 300 µL of elution buffer (10 mM Tris-HCl pH 8, 150 mM NaCl, 0.1% Triton x-100, 1 mM DTT) during 3 h at 4 °C. Eluted proteins were precipitated with TCA-DOC/Acetone, resuspended in 20 µL of keratin-free Laemmli buffer and concentrated and desalted by a short run SDS-PAGE. Two bands containing all eluted proteins for the experiment and for the control were excised from the gel and processed for LC/ESI-MS/MS and protein identification using the Mascot software at the proteomics facility of the Spanish National Centre for Biotechnology (CNB-CSIC).

### Analysis of Scramb1-A oligomerization

*UAS-scramb1-A-V5* and *UAS-scramb1-A-ProtA* were coexpressed in salivary glands using the *AB1-Gal4* driver. For the control, only *UAS-scramb1-A-V5* was expressed. 200 salivary glands were dissected for each genotype and lysates prepared and cleared as described in the co-immunoprecipitation section. The lysates (200 µL) were incubated with 25 µL of 50% slurry Epoxi Dynabeads M-270 conjugated to rabbit IgGs during 30 min at room temperature. The matrix was washed 6 times with 200 µL of lysis buffer and eluted in 20 µL of Laemmli buffer at 95 °C. Lysates (10%) and elution fractions were analyzed by western blot using anti-V5 antibody, which detects both, Scramb1-A-V5 and Scramb1-A-ProtA, since ProtA still binds with moderate affinity to IgGs after being transferred to the membrane.

### Antibodies

The following antibodies were used: mouse anti-V5 (Invitrogen, #46–0705), guinea pig anti-Duf extracellular [10], rabbit anti-Pyd [36], mouse anti-Pyd (DSHB, PYD2), chicken anti-Sns [78], rabbit anti-pSrc64 [79], rat anti-Cubn [80], rabbit anti-Ubiquitin (Sigma, #U5379), mouse anti-Myc (DSHB, 9E 10), rat anti-HA (Roche, high affinity #11,867,423,001), mouse anti-RFP (Chromotek, 6G6), rabbit anti-Past1 [48], rabbit anti-Amph [81], mouse anti-β-Galactosidase (Promega, #Z378A).

### Immunohistochemistry and in situ hybridization

Garland cells of the different genotypes analyzed were dissected in PBS from wandering third instar larvae or from first instar larvae (0–2 h after hatching), as indicated, kept at 4 °C and fixed by incubation in 0.7% NaCl, 0.005% Triton X-100 at 95 °C for 10 s. After a short wash in PBS, cells were permeabilized in PBS containing 0.3% Triton X-100 (PBT), blocked in PBT supplemented with 1% BSA for one hour and incubated overnight at 4 °C with the corresponding primary antibodies dissolved in PBT-BSA. After three washes in PBT for a total of one hour, cells were similarly incubated with the corresponding fluorescent secondary antibodies for 2 h at room temperature, washed and tissues mounted in 90% glycerol, 20 mM Tris-HCl pH 8.0 and 0.5% N-propyl gallate. Immunohistochemistry of salivary glands and imaginal discs was performed using a similar protocol, except that tissues were fixed in 4% formaldehyde in PBS for 40 min. Images were obtained in a Zeiss LSM800 confocal system or in an Olympus SpinSR10 confocal spinning disk microscope. Images taken at super-resolution were obtained using Olympus super-resolution technology, with a theoretical maximal spatial resolution of 120 nm, in the SpinSR10 system and processed by deconvolution using Huygens 19.10 software.

Intensity profiles were generated using the Fiji plugin “multichannel plot profiler” from the BAR collection. 10–17 profiles, 6 μm in length and 1.7 μm wide, were acquired across the plasma membrane from 4 to 7 cells. The data was processed and plotted using the python libraries pandas and Vega-Altair. Error bands display the confidence interval of the mean.

Pearson’s colocalization coefficients were calculated with the Fiji plugin JACoP, BIO Version.

In situ hybridization of embryos, garland cells and pericardial cells was performed as in [36] using RNA probes prepared from plasmids GM13876 and GH10494 [75], corresponding to *scramb1* and *scramb2* cDNAs respectively, linearized at the 5’ ends and transcribed using SP6 RNA polymerase using Roche DIG RNA labelling kit.

### Electron microscopy and immunogold labelling

Samples for electron microscopy were fixed in 4% formaldehyde and 2% glutaraldehyde in phosphate buffer pH 7.4 (PB) for 2 h at room temperature, washed and post-fixed in 1% osmium tetroxide in water at 4 °C for 1 h. After additional washes, nephrocytes were treated with 0.15% tannic acid for 1 min, followed by a wash and staining with 2% uranyl acetate in water in the dark for 1 h. Subsequently, the samples were washed, stepwise dehydrated in acetone and embedded in TAAB 812 epoxy resin. Ultrathin sections were obtained using an ultramicrotome Ultracut E (Leica) at the CBMSO electron microscopy facility and stained with 2% uranyl acetate for 7 min and lead citrate for 2 min. Imaging was performed in a JEM1400 Flash electron microscope coupled to a CMOS GATAN Oneview camera at 80 kV.

For post-embedding immunogold labelling of garland cells, the samples were fixed in 4% formaldehyde in PB at room temperature for 2 h, followed by overnight incubation at 4 °C. Glutaraldehyde was omitted to preserve V5 epitope immunoreactivity. The samples were pre-embedded in 10% gelatin to facilitate handling, cryoprotected in 30% glycerol, processed by plunge-freezing in liquid ethane and freeze-substitution in methanol containing 0.5% uranyl acetate using an AFS apparatus (Leica), infiltrated with Lowicryl HM20 at -40 °C, and polymerized with ultraviolet light. Ultrathin sections were washed, blocked in 10% fetal bovine serum in 30 mM Tris pH 8.2 and 150 mM NaCl (TBS), and then incubated in anti-V5 antibody at a 1:40 dilution in TBS with 5% fetal bovine serum for 1 h. After washes, the sections were incubated in rabbit anti-mouse antibody at a 1:100 dilution in the same buffer for 45 min, followed by washes and incubation in Protein A gold 15 nm (Cell Microscopy Core) at a 1:50 dilution for 1 h. After additional washes, the sections were stained with uranyl acetate and lead citrate. As a control, ultrathin sections were processed in a similar manner, but excluding the anti-V5 antibody. The immunogold labelling was performed at the CBMSO electron microscopy facility. Quantitative analysis of 21 images obtained from three different cells was conducted following the methods described in Mayhew, 2007 [82]. This involved comparing the frequency distribution of observed gold particles with a randomly generated distribution of particles in two pre-defined cell compartments: SDs and cytoplasm. A chi-square test yielded a total chi-square value of 12,880, which corresponds to a highly significant *p* value (< 0.00001). The SD compartment exhibited an enrichment of 101 times compared to a random distribution and contributed the majority of the chi-square value. Image analysis was carried out using the Fiji software.

### Quantitative analysis of genetic interactions

Crosses were kept at 25 ºC except otherwise indicated. Nephrocytes of the different genotypes analyzed were dissected and stained with anti-Duf, anti-Pyd and DAPI. Z-stacks, each containing one cluster of garland cells, were captured in a SpinSR10 system. For each visible nephrocyte in the stacks we generated one Z-projection comprising from 5 to 12 cortical sections from the cell region closer to the microscope objective, using the Fiji software. All the Z-projections for each genotype (*n* = 87–193) were combined in a single canvas and visually classified in four different categories based on the proportion of the imaged nephrocyte surface covered by SDs, identified by the Duf pattern. The categories were defined to minimize subjectivity, resulting in two different persons producing similar classifications. Categories are: (0) No visible SD strands; (1) SD strands covering less than 10% of the visible surface; (2) More SDs than 1 and still some regions not covered by SDs; (3) SDs cover all visible surface. Examples of these categories are shown in Fig. S7. Plots were generated using the Vega-Altair library in Python. Statistical significance was evaluated by a nonparametric Mann-Whitney U test with correction for tied ranks, using the SciPy library. Similar significance levels were obtained by the Cochran-Armitage test for trend, sensitive to upward or downward trends in ordered categories.

### Electronic supplementary material

Below is the link to the electronic supplementary material.

Supplementary Material 1

Supplementary Material 2

Supplementary Material 3

Supplementary Material 4

Supplementary Material 5

Supplementary Material 6

Supplementary Material 7

Supplementary Material 8

Supplementary Material 9

Supplementary Material 10

## Data Availability

The dataset generated in this study is included as Supplementary Material. We analyzed the previously published dataset: Sue A. Krause, Gayle Overend, Julian A. T. Dow, David P. Leader, 2021, “Drosophila Garland Cell Transcriptome”, https://www.ebi.ac.uk/ena/browser/view/PRJEB48667, European Nucleotide Archive; PRJEB48667.

## References

[1] Patrakka J, Tryggvason K (2009). New insights into the role of podocytes in proteinuria. Nat Rev Nephrol.

[2] Scott RP, Quaggin SE (2015). The cell biology of renal filtration. J Cell Biol.

[3] Tryggvason K, Patrakka J, Wartiovaara J (2006). Hereditary Proteinuria syndromes and mechanisms of Proteinuria. N Engl J Med.

[4] Barletta G-M, Kovari IA, Verma RK (2003). Nephrin and Neph1 co-localize at the podocyte foot process Intercellular Junction and Form cis Hetero-oligomers. J Biol Chem.

[5] Gerke P, Huber TB, Sellin L (2003). Homodimerization and heterodimerization of the glomerular podocyte proteins nephrin and NEPH1. JASN.

[6] Kestilä M, Lenkkeri U, Männikkö M (1998). Positionally cloned gene for a Novel glomerular protein—Nephrin—Is mutated in congenital nephrotic syndrome. Mol Cell.

[7] Holzman LB, John ST, Kovari PL (1999). Nephrin localizes to the slit pore of the glomerular epithelial cell. Kidney Int.

[8] Donoviel DB, Freed DD, Vogel H (2001). Proteinuria and Perinatal Lethality in mice lacking NEPH1, a Novel protein with homology to NEPHRIN. Mol Cell Biol.

[9] Grahammer F, Schell C, Huber TB (2013). The podocyte slit diaphragm–from a thin grey line to a complex signalling hub. Nat Rev Nephrol.

[10] Weavers H, Prieto-Sánchez S, Grawe F (2009). The insect nephrocyte is a podocyte-like cell with a filtration slit diaphragm. Nature.

[11] Zhuang S, Shao H, Guo F (2009). Sns and Kirre, the Drosophila orthologs of Nephrin and Neph1, direct adhesion, fusion and formation of a slit diaphragm-like structure in insect nephrocytes. Development.

[12] Tutor AS, Prieto-Sanchez S, Ruiz-Gomez M (2014). Src64B phosphorylates dumbfounded and regulates slit diaphragm dynamics: Drosophila as a model to study nephropathies. Development.

[13] Koehler S, Huber TB (2023). Insights into human kidney function from the study of Drosophila. Pediatr Nephrol.

[14] Arif E, Wagner MC, Johnstone DB (2011). Motor protein Myo1c is a podocyte protein that facilitates the Transport of Slit Diaphragm Protein Neph1 to the Podocyte membrane. Mol Cell Biol.

[15] Simons M, Schwarz K, Kriz W (2001). Involvement of lipid rafts in Nephrin Phosphorylation and Organization of the Glomerular Slit Diaphragm. Am J Pathol.

[16] Verma R, Wharram B, Kovari I (2003). Fyn binds to and phosphorylates the kidney Slit Diaphragm Component Nephrin*. J Biol Chem.

[17] Yu C, Zhang H, Liu S (2023). Flot2 acts as a novel mediator of podocyte injury in proteinuric kidney disease. Int J Biol Sci.

[18] Qin X-S, Tsukaguchi H, Shono A (2009). Phosphorylation of Nephrin triggers its internalization by Raft-Mediated Endocytosis. JASN.

[19] Huber TB, Simons M, Hartleben B (2003). Molecular basis of the functional podocin–nephrin complex: mutations in the NPHS2 gene disrupt nephrin targeting to lipid raft microdomains. Hum Mol Genet.

[20] Huber TB, Schermer B, Müller RU (2006). Podocin and MEC-2 bind cholesterol to regulate the activity of associated ion channels. PNAS.

[21] Schwarz K, Simons M, Reiser J (2001). Podocin, a raft-associated component of the glomerular slit diaphragm, interacts with CD2AP and nephrin. J Clin Invest.

[22] Lang K, Milosavljevic J, Heinkele H (2022). Selective endocytosis controls slit diaphragm maintenance and dynamics in Drosophila nephrocytes. eLife.

[23] Carrasco-Rando M, Culi J, Campuzano S, Ruiz-Gómez M (2023). An acytokinetic cell division creates PIP2-enriched membrane asymmetries leading to slit diaphragm assembly in Drosophila nephrocytes. Development.

[24] Gass MM, Borkowsky S, Lotz M-L (2022). PI(4,5)P2 controls slit diaphragm formation and endocytosis in Drosophila nephrocytes. Cell Mol Life Sci.

[25] Tomancak P, Berman BP, Beaton A (2007). Global analysis of patterns of gene expression during Drosophila embryogenesis. Genome Biol.

[26] Zhou Q, Zhao J, Stout JG (1997). Molecular cloning of human plasma membrane phospholipid scramblase. A protein mediating transbilayer movement of plasma membrane phospholipids. J Biol Chem.

[27] Sims PJ, Wiedmer T (2001). Unraveling the mysteries of Phospholipid Scrambling. Thromb Haemost.

[28] Frasch SC, Henson PM, Nagaosa K (2004). Phospholipid flip-Flop and Phospholipid Scramblase 1 (PLSCR1) co-localize to Uropod rafts in Formylated Met-Leu-Phe-stimulated neutrophils. J Biol Chem.

[29] Sahu SK, Gummadi SN, Manoj N, Aradhyam GK (2007). Phospholipid scramblases: an overview. Arch Biochem Biophys.

[30] Bateman A, Finn RD, Sims PJ (2009). Phospholipid scramblases and Tubby-like proteins belong to a new superfamily of membrane tethered transcription factors. Bioinformatics.

[31] Ory S, Ceridono M, Momboisse F (2013). Phospholipid Scramblase-1-Induced lipid reorganization regulates compensatory endocytosis in neuroendocrine cells. J Neurosci.

[32] Cheshenko N, Pierce C, Herold BC (2018). Herpes simplex viruses activate phospholipid scramblase to redistribute phosphatidylserines and akt to the outer leaflet of the plasma membrane and promote viral entry. PLoS Pathog.

[33] Acharya U, Edwards MB, Jorquera RA (2006). Drosophila melanogaster scramblases modulate synaptic transmission. J Cell Biol.

[34] Krause SA, Overend G, Dow JAT, Leader DP (2021) FlyAtlas 2 in 2022: enhancements to the Drosophila melanogaster expression atlas. 10.1093/nar/gkab971. Nucleic Acids Research gkab971 10.1093/nar/gkab971 PMC8728208 34718735

[35] McGuire SE, Le PT, Osborn AJ (2003). Spatiotemporal Rescue of Memory Dysfunction in Drosophila. Science.

[36] Carrasco-Rando M, Prieto-Sánchez S, Culi J et al (2019) A specific isoform of Pyd/ZO-1 mediates junctional remodeling and formation of slit diaphragms. J Cell Biol Jcb 201810171. 10.1083/jcb.201810171 10.1083/jcb.201810171 PMC6605796 31171632

[37] Branon TC, Bosch JA, Sanchez AD (2018). Efficient proximity labeling in living cells and organisms with TurboID. Nat Biotechnol.

[38] Yano H, Yamamoto-Hino M, Awano W (2012). Identification of Proteasome Components required for apical localization of Chaoptin using Functional Genomics. J Neurogenet.

[39] Kato N, Nakanishi M, Hirashima N (2002). Transbilayer asymmetry of phospholipids in the plasma membrane regulates exocytotic release in mast cells. Biochemistry.

[40] Zhou Q, Sims PJ, Wiedmer T (1998). Identity of a conserved motif in Phospholipid Scramblase that is required for Ca2+-Accelerated Transbilayer Movement of Membrane Phospholipids. Biochemistry.

[41] Stuermer CAO, Lang DM, Kirsch F (2001). Glycosylphosphatidyl Inositol-anchored proteins and fyn kinase assemble in Noncaveolar plasma membrane microdomains defined by Reggie-1 and – 2. MBoC.

[42] Hoehne M, Gert de Couet H, Stuermer CAO, Fischbach K-F (2005). Loss- and gain-of-function analysis of the lipid raft proteins Reggie/Flotillin in Drosophila: they are posttranslationally regulated, and misexpression interferes with wing and eye development. Mol Cell Neurosci.

[43] Sun J, Nanjundan M, Pike LJ (2002). Plasma membrane phospholipid scramblase 1 is enriched in lipid rafts and interacts with the epidermal growth factor receptor. Biochemistry.

[44] Zhao J, Zhou Q, Wiedmer T, Sims PJ (1998). Palmitoylation of Phospholipid Scramblase is required for normal function in promoting Ca2+-Activated Transbilayer Movement of Membrane Phospholipids. Biochemistry.

[45] Strassburger K, Kang E, Teleman AA (2019). Drosophila ZDHHC8 palmitoylates scribble and Ras64B and controls growth and viability. PLoS ONE.

[46] Wiedmer T, Zhao J, Nanjundan M, Sims PJ (2003). Palmitoylation of Phospholipid Scramblase 1 Controls its distribution between Nucleus and plasma membrane. Biochemistry.

[47] Naslavsky N, Caplan S (2011). EHD proteins: key conductors of endocytic transport. Trends Cell Biol.

[48] Olswang-Kutz Y, Gertel Y, Benjamin S (2009). Drosophila Past1 is involved in endocytosis and is required for germline development and survival of the adult fly. J Cell Sci.

[49] Pant S, Sharma M, Patel K (2009). AMPH-1/Amphiphysin/Bin1 functions with RME-1/Ehd1 in endocytic recycling. Nat Cell Biol.

[50] Koles K, Messelaar EM, Feiger Z (2015). The EHD protein Past1 controls postsynaptic membrane elaboration and synaptic function. Mol Biol Cell.

[51] Hazel JR (1995). Thermal adaptation in biological membranes: is homeoviscous adaptation the explanation?. Annu Rev Physiol.

[52] Kocylowski MK, Aypek H, Bildl W (2022). A slit-diaphragm-associated protein network for dynamic control of renal filtration. Nat Commun.

[53] Hermle T, Braun DA, Helmstädter M (2017). Modeling monogenic human nephrotic syndrome in the Drosophila Garland Cell Nephrocyte. J Am Soc Nephrol.

[54] Francis VG, Mohammed AM, Aradhyam GK, Gummadi SN (2013). The single C-terminal helix of human phospholipid scramblase 1 is required for membrane insertion and scrambling activity. FEBS J.

[55] Luévano-Martínez LA, Kowaltowski AJ (2017). Topological characterization of the mitochondrial phospholipid scramblase 3. FEBS Lett.

[56] Kametaka S, Shibata M, Moroe K (2003). Identification of Phospholipid Scramblase 1 as a Novel Interacting Molecule with β-Secretase (β-Site amyloid precursor protein (APP) cleaving enzyme (BACE)). J Biol Chem.

[57] Rayala S, Francis VG, Sivagnanam U, Gummadi SN (2014). N-terminal proline-rich domain is required for scrambling activity of human phospholipid scramblases. J Biol Chem.

[58] Sun J, Zhao J, Schwartz MA (2001). c-Abl tyrosine kinase binds and phosphorylates Phospholipid Scramblase 1. J Biol Chem.

[59] Xu D, Jiang W, Wu L (2023). PLSCR1 is a cell-autonomous defence factor against SARS-CoV-2 infection. Nature.

[60] Djenoune L, Tomar R, Dorison A (2021). Autonomous Calcium signaling in human and zebrafish podocytes controls kidney filtration barrier morphogenesis. J Am Soc Nephrol.

[61] Sivakumar S, Miellet S, Clarke C, Hartley PS (2022). Insect nephrocyte function is regulated by a store operated calcium entry mechanism controlling endocytosis and amnionless turnover. J Insect Physiol.

[62] Resh MD (2006) Palmitoylation of Ligands, Receptors, and Intracellular Signaling Molecules. Science’s STKE 2006:re14–re14. 10.1126/stke.3592006re14 10.1126/stke.3592006re14 17077383

[63] Sellin L, Huber TB, Gerke P (2003). NEPH1 defines a novel family of podocin-interacting proteins. FASEB J.

[64] Kann M, Ettou S, Jung YL (2015). Genome-wide analysis of Wilms’ Tumor 1-Controlled gene expression in Podocytes reveals Key Regulatory mechanisms. JASN.

[65] Baelde HJ, Eikmans M, Doran PP (2004). Gene expression profiling in glomeruli from human kidneys with diabetic nephropathy. Am J Kidney Dis.

[66] Cooley L, Kelley R, Spradling A (1988). Insertional mutagenesis of the Drosophila genome with single P elements. Science.

[67] Bellen HJ, Levis RW, Liao G (2004). The BDGP gene disruption project: single transposon insertions Associated with 40% of Drosophila genes. Genetics.

[68] Kondo S, Ueda R (2013). Highly improved gene targeting by germline-specific Cas9 expression in Drosophila. Genetics.

[69] Djiane A, Shimizu H, Wilkin M (2011). Su(dx) E3 ubiquitin ligase–dependent and –independent functions of Polychaetoid, the Drosophila ZO-1 homologue. J Cell Biol.

[70] Razzaq A, Robinson IM, McMahon HT (2001). Amphiphysin is necessary for organization of the excitation–contraction coupling machinery of muscles, but not for synaptic vesicle endocytosis in Drosophila. Genes Dev.

[71] Bischof J, Maeda RK, Hediger M (2007). An optimized transgenesis system for Drosophila using germ-line-specific φC31 integrases. PNAS.

[72] Bischoff M, Gradilla A-C, Seijo I (2013). Cytonemes are required for the establishment of a normal hedgehog morphogen gradient in Drosophila epithelia. Nat Cell Biol.

[73] Asha H, Nagy I, Kovacs G (2003). Analysis of ras-Induced Overproliferation in Drosophila Hemocytes. Genetics.

[74] Tanimoto H, Itoh S, ten Dijke P, Tabata T (2000). Hedgehog creates a gradient of DPP Activity in Drosophila Wing Imaginal Discs. Mol Cell.

[75] Stapleton M, Carlson J, Brokstein P et al (2002) A Drosophila full-length cDNA resource. Genome Biol 3:research0080.1. 10.1186/gb-2002-3-12-research0080 10.1186/gb-2002-3-12-research0080 PMC151182 12537569

[76] Veraksa A, Bauer A, Artavanis-Tsakonas S (2005). Analyzing protein complexes in Drosophila with tandem affinity purification–mass spectrometry. Dev Dyn.

[77] Oeffinger M, Wei KE, Rogers R (2007). Comprehensive analysis of diverse ribonucleoprotein complexes. Nat Methods.

[78] Hochapfel F, Denk L, Mendl G et al (2017) Distinct functions of Crumbs regulating slit diaphragms and endocytosis in Drosophila nephrocytes. Cell Mol Life Sci 1–14. 10.1007/s00018-017-2593-y 10.1007/s00018-017-2593-y PMC11107785 28717874

[79] O’Reilly AM, Ballew AC, Miyazawa B (2006). Csk differentially regulates Src64 during distinct morphological events in Drosophila germ cells. Development.

[80] Atienza-Manuel A, Castillo-Mancho V, De Renzis S (2021). Endocytosis mediated by an atypical CUBAM complex modulates slit diaphragm dynamics in nephrocytes. Development.

[81] Zelhof AC, Bao H, Hardy RW (2001). Drosophila Amphiphysin is implicated in protein localization and membrane morphogenesis but not in synaptic vesicle endocytosis. Development.

[82] Mayhew TM (2007) Quantitative Immunoelectron Microscopy. ElectronMicroscopy, second. Humana, pp 309–329 10.1007/978-1-59745-294-6_15 17656757

