# Supplementary material for: Phospholipid scramblase 1: an essential component of the nephrocyte slit diaphragm
Source: Cell Mol Life Sci. 2024 Jun 15;81(1):261. doi: 10.1007/s00018-024-05287-z (PMC11335299; doi:10.1007/s00018-024-05287-z)
Supplement: Supplementary file 1 — Supplementary Material 1 [file 18_2024_5287_MOESM1_ESM.docx]

**Figure S1. (A-B)** *scramb1* is expressed in the primordium of the garland cells in a wild-type stage 12 embryo (A, arrow) and in larval pericardial nephrocytes (B) as detected by in situ hybridization. **(C)** Genomic map of a chromosome 3L segment depicting scramb1-A and scramb1-B transcripts, the location of the transposon EY07744 used to generate the allele *scramb1^43^* by its imprecise excision and the genomic region deleted in this allele. The location of the oligonucleotide used for the reverse trascription of RNA from wild-type and *scramb1^43^* animals (red arrows) as well as the oligonucleotide pairs (black arrows) used to amplify from the reverse transcription reaction all *scramb1* isoforms (PCR B) or to specifically amplify the scramb1-A transcript (PCR A) are also shown. **(D)** PCR products obtained from the RT-PCR reactions described in C. Arrows point to the specific PCR products. MW: 4367, 2898, 2498, 2201, 1933, 1331, 1150, 759, 611 base pairs. **(E)** in situ hybridization to detect *scramb1* mRNA in wild-type and in *scramb1^43^* larval garland nephrocytes. **(F)** Schematic representation illustrating the protein domain architecture and the location of conserved putative Ca^2+^ binding and palmitoylation sites within Scramb1-A and -B isoforms as well as in its homologs, human PLSCR1, and *Drosophila* Scramb2. An arrow in Scramb2 denotes the positions of the frameshift mutations in *scramb2^V3^* (Asp-17) and *scramb2^V6^* (Gln-16) alleles. **(G)** In situ hybridization to detect *scramb2* expression in a wild-type stage 16 *Drosophila* embryo. *scramb2* is enriched in the garland cells (arrow).

**Figure S2. (A-B)** Distribution of SDs, detected by the coexpression of Duf and Pyd, in wild-type (A) and *scramb1^43^* (B) first instar larvae. **(C-E')** Immunostaining of *scramb2^V3^* (C and C'), *scramb2^V6^*, *scramb1^43^* double mutant (D and D'), and *scramb1^43^* nephrocytes from third instar larvae expressing *UAS-scramb2-HA* driven by *pros-gal4* (E and E') to show the distribution of Duf and Pyd proteins in medial (C, D and E) and cortical (C', D' and E') planes. The *scramb2^V3^* mutant exhibits no SD phenotype, while the double mutant *scramb2^V6^*, *scramb1^43^* phenotype is equivalent to that of *scramb1^43^* (Fig. 2, B and B'), with scant SDs and abundant cortical foci containing Pyd but not Duf. Expression of *scramb2-HA* fails to rescue SD formation in *scramb1^43^* cells. C-E' shown at the same magnification. **(F)** Ultrastructure of the cortical region of a wild-type nephrocyte, to illustrate an abundance of SDs (arrows) decorating the plasma membrane. **(G)** Ultrastructure of the cortical region of a *scramb1^43^* nephrocyte, showing the presence of occasional SD structures (red arrows). (**H**) Quantitation of SDs and cortical electron-dense plaques in TEM micrographs of wild-type, *scramb1^43^*, and *scramb1^43^* nephrocytes rescued by temporally controlled expression of *UAS-scramb1-A-V5* driven by *pros-gal4* (rescue), as depicted in I. The number of features scored by µm of plasma membrane length is plotted (n=5 cells per genotype). **(I)** Immunostaining of *scramb1^43^* nephrocytes rescued by the expression of *UAS-scramb1-A-V5* driven by *pros-Gal4* to show the distribution of Duf, Pyd and Scramb1-A-V5, as indicated. The temporal expression of the transgene was controlled using the TARGET technology. The complete genotype is described in the Methods section. Animals were maintained at the permissive temperature (29 °C) for the 96 hours after egg deposition, followed by a switch to the restrictive temperature (18 °C) for the 72 hours previous to dissection to prevent the accumulation of ectopic Scramb1-A-V5. Duf, Pyd and Scramb1-A-V5 colocalize in the cortical region. This genotype and conditions are identical as in Fig. 2, G and H.

**Figure S3.** Additional time-points and medial sections corresponding to the time-course analysis of SD induction by Scramb1-A. Immunostaining of *scramb1^43^* or *scramb1^43^* nephrocytes rescued by the expression of *UAS-scramb1-A-V5* for increasing periods of time (0, 6, 12 and 18 hours, as indicated) using the TARGET technology. The distribution of Scramb1-A-V5 (anti-V5 antibody), Sns, Pyd, Duf and phospho-Src64B are shown, as indicated. At 0 hours, cortical foci containing Pyd and lacking Duf are visible (white arrowheads). After 6 hours of expression, Scramb1-A-V5 starts to be detectable in the cortex simultaneously with the accumulation of low levels of Duf in foci that express Pyd. At the 12 hours time-point, short rods containing Sns, Duf and the active, phosphorylated form of Src64B are visible (yellow arrowheads). Medial sections corresponding to the 0, 12 and 18 hours time-points are also shown.

**Figure S4.** **(A)** S2 cells co-transfected with Scramb1-A-V5 and Duf-GFP. Duf-GFP, detected by autofluorescence, localizes to the cell membrane and, through homophilic interactions, induces the aggregation of S2 cells, stabilizing Duf-GFP at cell-cell contact region (arrow). In contrast, Scramb1-A-V5 (anti-V5 antibody) is uniformly distributed in the plasma membrane, indicating that Duf does not recruit Scramb1-V5 in S2 cells. **(B-C)** Immunostaining of salivary glands coexpressing *UAS-scramb1-A-V5* and *UAS-pyd-PΔCC* (B) or *UAS-pyd-P* (C) driven by *AB1-Gal4*. Scramb1-A-V5 colocalizes with Pyd-P and Pyd-PΔCC in the plasma membrane. **(D)** Cortical section of *duf^sps1^* nephrocytes expressing *UAS-scramb1-A-V5* (*pros-Gal4*), showing the colocalization of Scramb1-A-V5, Pyd and Sns in cortical foci. **(E-F)** Immunostaining of wing imaginal discs expressing *UAS-scramb1-A-V5* (E) or *UAS-scramb1-B-V5* (F) in the posterior compartment driven by *hh-Gal4*. Scramb1-B protein is stable in the epithelial tissue of imaginal discs, although it accumulates at lower levels than the Scramb1-A isoform. Filamentous actin is revealed by phalloidin staining. **(G)** Immunostaining of garland nephrocytes expressing *UAS-scramb1-B-V5* and an interfering RNA to silence the proteasome subunit *Prosβ3*. Scramb1-B-V5 (anti-V5 antibody) accumulates in aggresomes and does not colocalize with cortical Pyd. **(H-I)** *scramb1^43^* nephrocytes expressing the chimeric protein *UAS-Spro-scramb2-V5* displayed at medial (H) and cortical (I) planes. There is no rescue of the *scramb1^43^* phenotype (compare with Fig. 2 B, B'), as shown by the distribution of Pyd and Duf. (B, C, G and H) Nuclei were labeled with DAPI, shown in blue.

**Figure S5. (A)** Alignment of the human PLSCR1 Ca^2+^ binding region with the homologous sequence in *Drosophila* Scramb1-A. Numbered residues were tested by mutagenesis. **(B-C')** Immunostaining of *scramb1^43^* nephrocytes rescued by the expression of the Ca^2+^ binding mutant variants Scramb1-A-A^D372A^-V5 (B-B') or Scramb1-A^F374A^-V5 (C-C') driven by *pros-Gal4*, shown at medial (B, C) and at higher magnification cortical (B', C') sections. Abundant SDs decorate the surface of the nephrocytes, as detected by Duf expression. Both Ca^2+^ binding variants (anti-V5 antibody) localize to SDs. **(D-E)** Immunostaining of *scramb1^43^* mutant nephrocytes (D) and *scramb1^43^* nephrocytes rescued by the expression of Scramb1-A-ProtA driven by *pros-Gal4* (E) using anti-Duf antibody which detects both Duf and Scramb1-A-ProtA due to the ProtA tag. The main images correspond to medial sections, and the inset in E shows a cortical section at a higher magnification. SDs can be observed in the rescued nephrocytes (E), but only scarce SDs are present in *scramb1^43^* (D). (B, C, D and E) Nuclei were labeled with DAPI. B, C and B', C' shown at the same magnification.

**Figure S6. (A-B')** Immunostaining of nephrocytes expressing Flo2-RFP driven by *sns-GCN-Gal4* in an otherwise wild-type background (A-A') or in *scramb1* silenced nephrocytes (B-B'). The distribution of Flo2-RFP (anti-RFP antibody) and Duf are shown as indicated. Flo2-RFP accumulates in the cortical region colocalizing with Duf in a wild-type background (A-A', arrows) but not in *scramb1* silenced nephrocytes (B-B', arrows). All panels at the same magnification. **(C-D')** Distribution of Scramb1-A-V5 (anti-V5 antibody, driven by *pros-Gal4*) and Pyd in nephrocytes of males hemizygous for the strong allele *Flo2^KG002110^* (D-D') and in the control heterozygous females (C-C'), shown at medial and at higher magnification cortical sections, as indicated. The distribution of Scramb1-A-V5 is not affected by *Flo2* loss of function. **(E-F)** Phenotype of *Past1^110.1^* mutants, showing partially aggregated nephrocytes and a distribution of SD strands, detected by the expression of Duf and Pyd, similar to the wild-type. Medial (E) and cortical (F) views.

**Figure S7.** Representative examples of nephrocytes corresponding to the indicated genotypes and conditions, utilized for the quantitation of phenotypes shown in Fig. 7 D and Fig. 8 C histograms. One example per category is displayed. The number of cells classified in each category and genotype/condition is provided. Each image results form a Z-projection of 5 to 12 cortical sections of nephrocytes stained for Duf and Pyd, as described in the Methods section. In some images, non-relevant cells are crossed out with white lines. Each nephrocyte was classified into one of four categories: (0): No visible SD strands in the imaged region; (1) SD strands covering less than 10% of the imaged region; (2) SDs covering a surface greater than 10% but still incomplete and (3) SD strands covering all the imaged region of the nephrocyte.

**Figure S8.** Model illustrating the dual role of Scramb1 in nephrocytes. (1) Scramb1 integrates into lipid raft microdomains via its palmitate adducts. Its ability to oligomerize and interact with the lipid raft-resident protein Flot2 and the adaptor protein Pyd facilitates the formation of a multiprotein platform crucial for SD assembly. This platform includes the adhesion molecules Sns and Duf, as well as the kinase Src64B. (2) Scramb1 engages in physical and/or genetic interactions with the C-terminal EHD protein Past1 and Amph, proteins involved in membrane remodeling processes such as membrane bending and scission. We propose that Scramb1 participates in membrane remodeling events during SD formation. These functions of Scramb1 are controlled by Ca^2+^ signaling.

**Table S1. List of proteins co-purifying with Scramb1-A-ProtA identified by mass spectrometry.** Only proteins absent in the control experiment, performed with empty matrix, are listed.
