# Supplementary figures and images for: Phospholipid scramblase 1: an essential component of the nephrocyte slit diaphragm
Source: Cell Mol Life Sci. 2024 Jun 15;81(1):261. doi: 10.1007/s00018-024-05287-z (PMC11335299; doi:10.1007/s00018-024-05287-z)

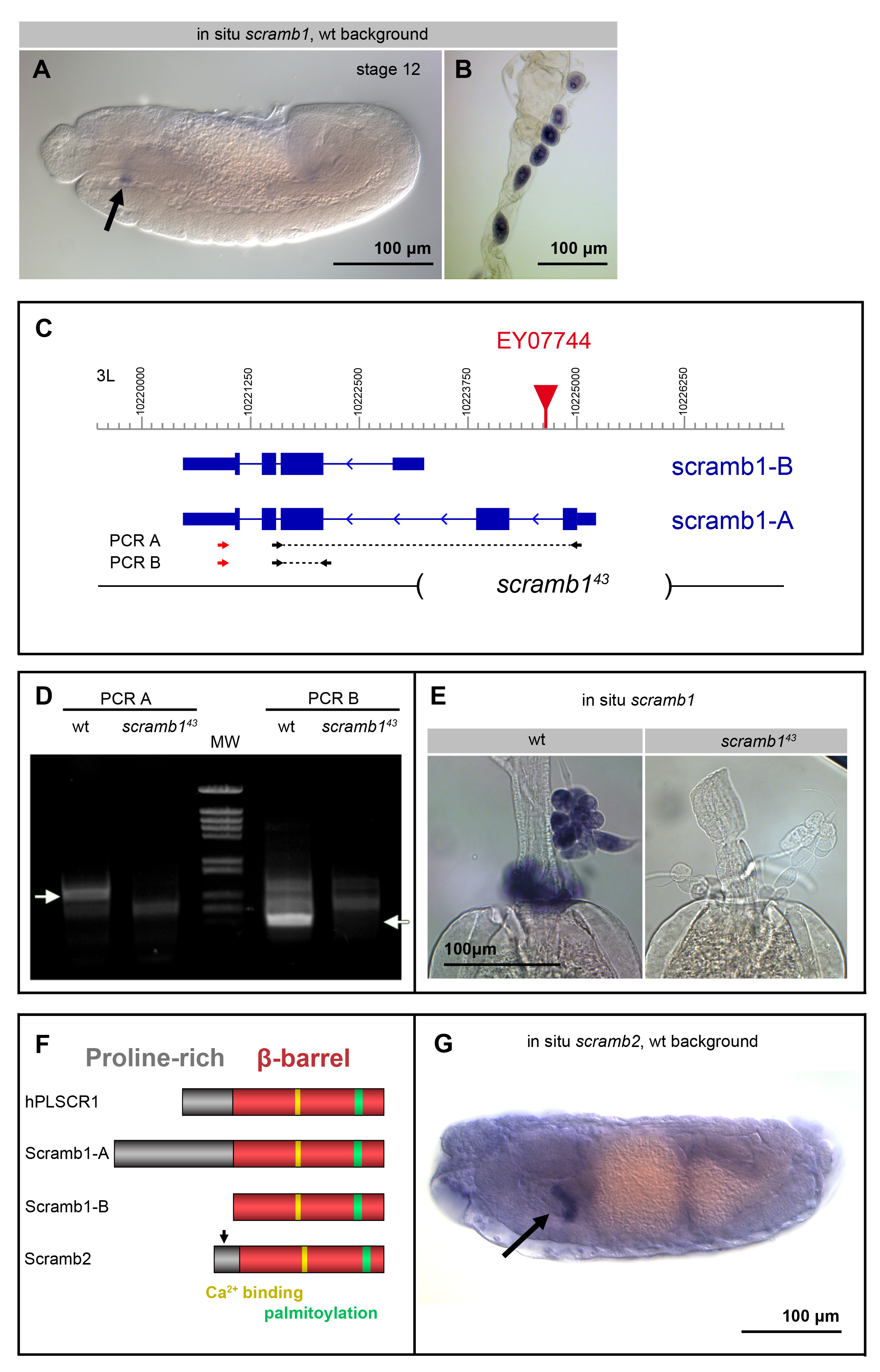

Supplement: Supplementary file 2 — Supplementary Material 2 [file 18_2024_5287_MOESM2_ESM.jpg]

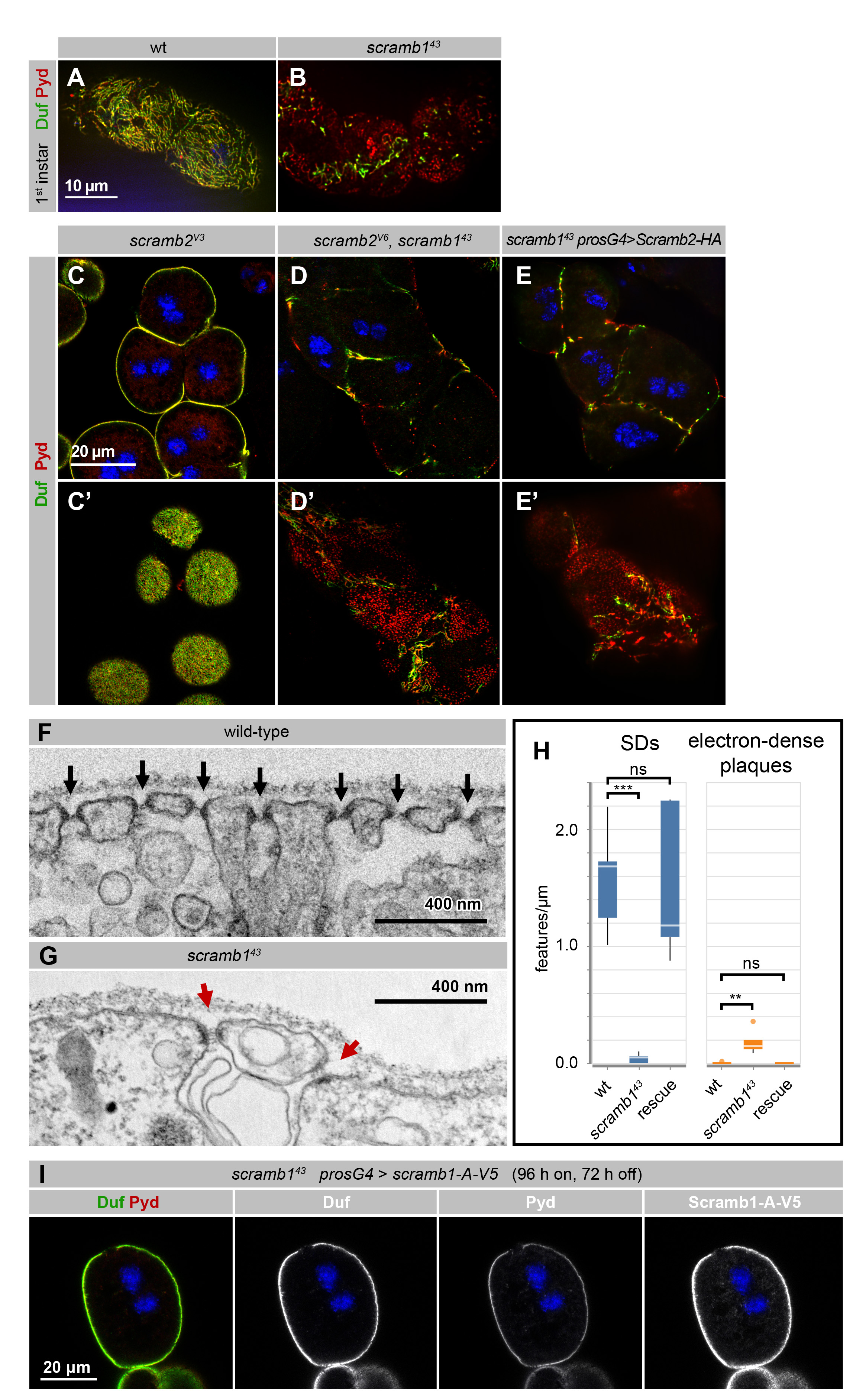

Supplement: Supplementary file 3 — Supplementary Material 3 [file 18_2024_5287_MOESM3_ESM.jpg]

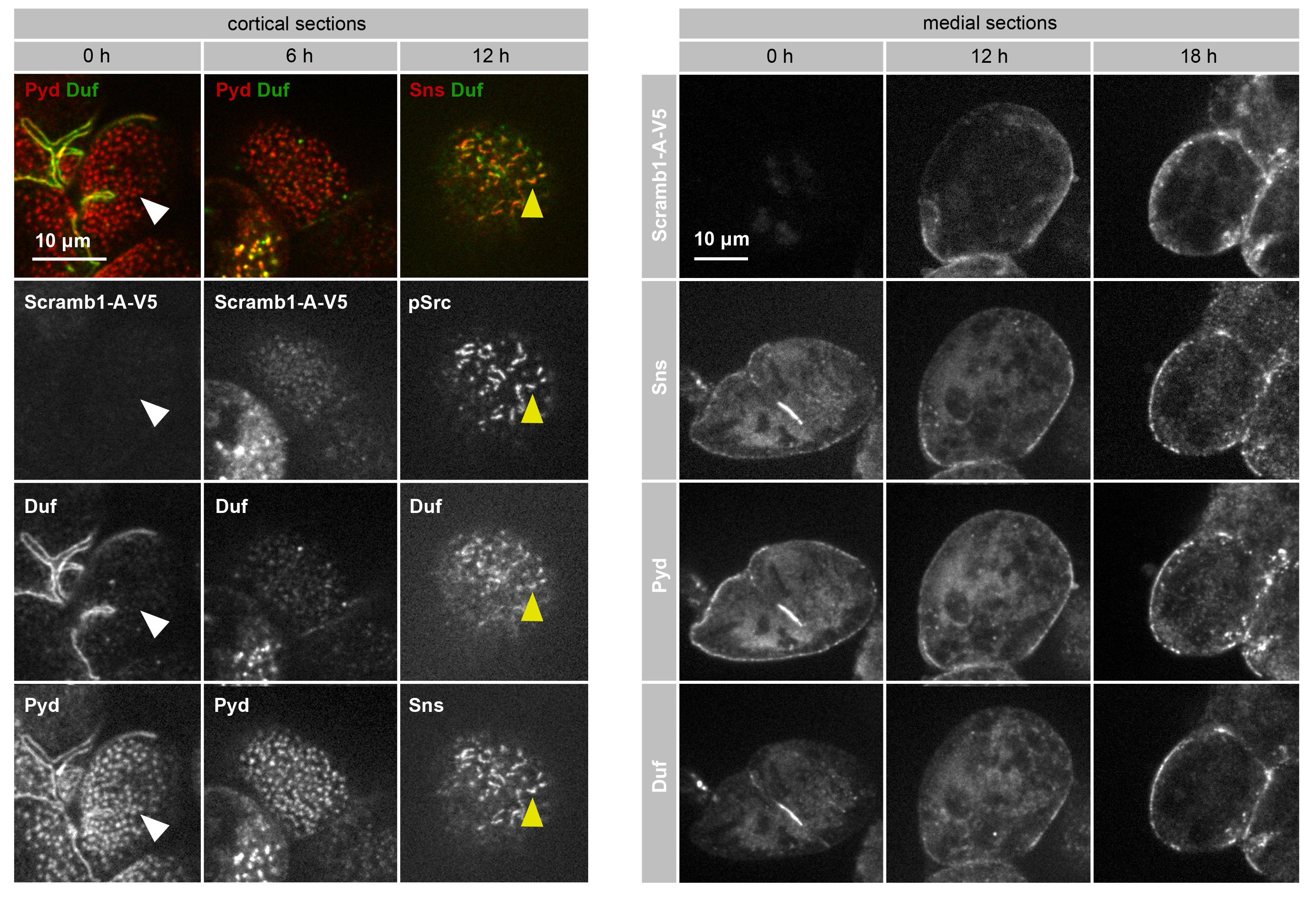

Supplement: Supplementary file 4 — Supplementary Material 4 [file 18_2024_5287_MOESM4_ESM.jpg]

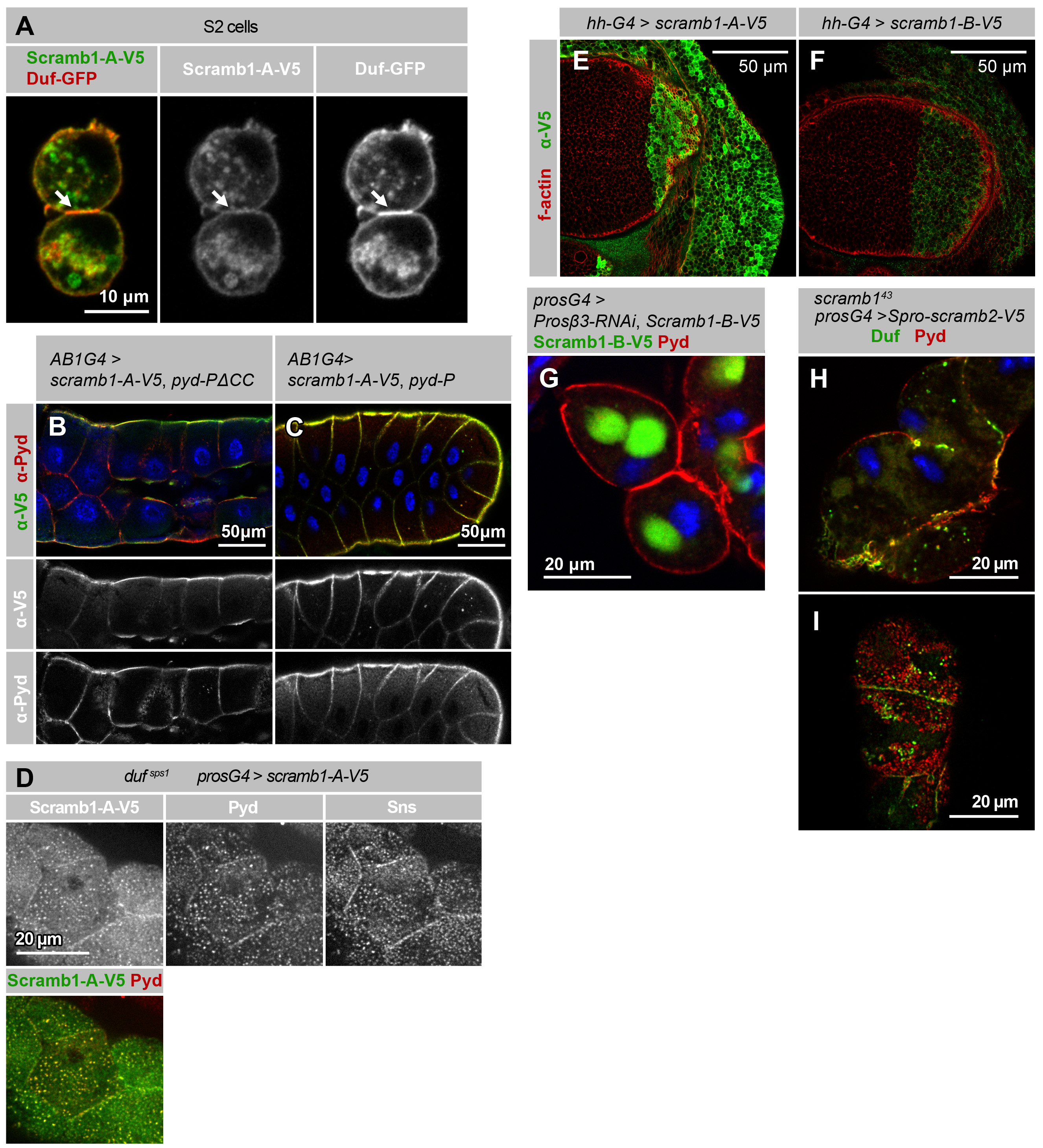

Supplement: Supplementary file 5 — Supplementary Material 5 [file 18_2024_5287_MOESM5_ESM.jpg]

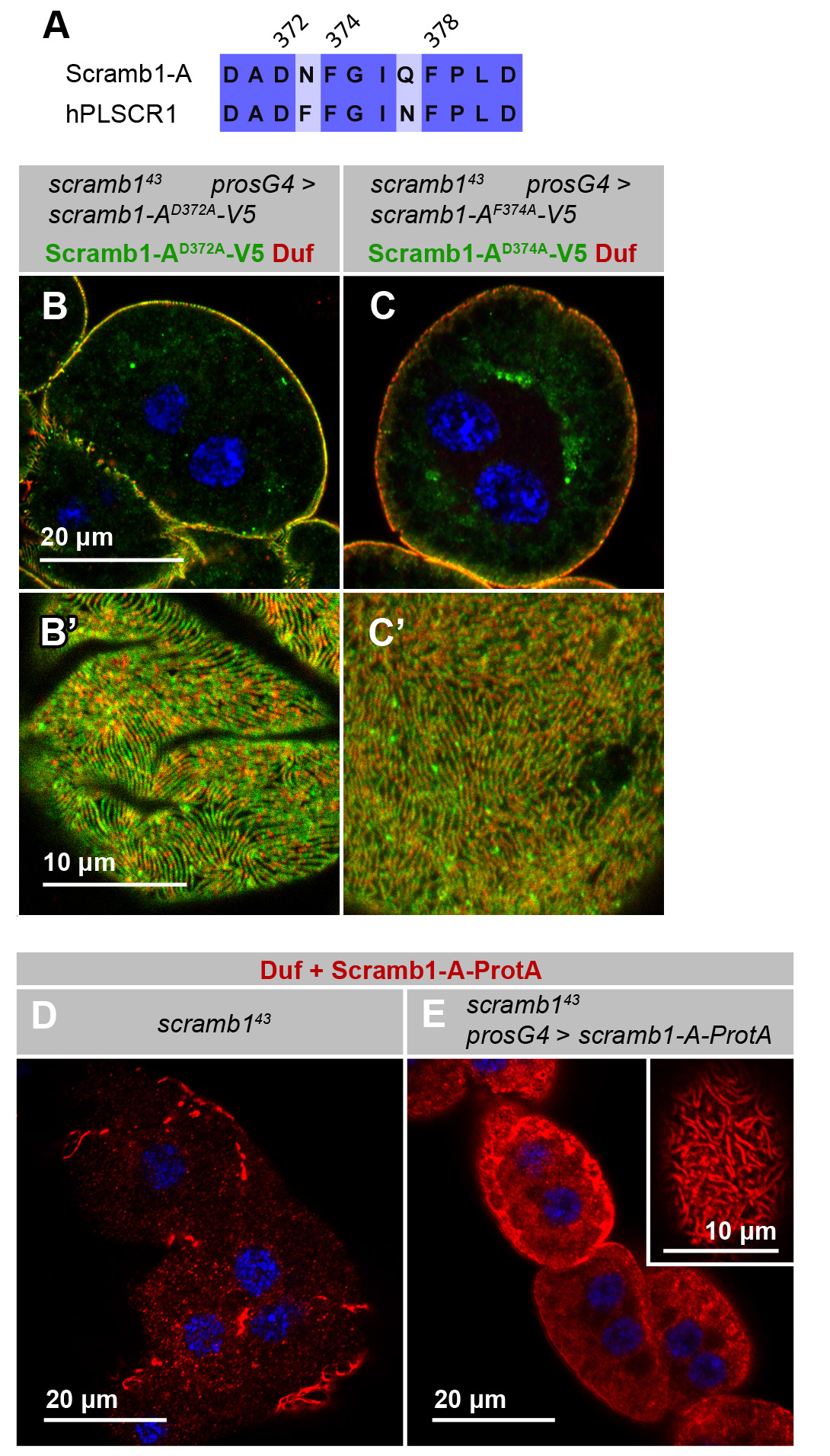

Supplement: Supplementary file 6 — Supplementary Material 6 [file 18_2024_5287_MOESM6_ESM.jpg]

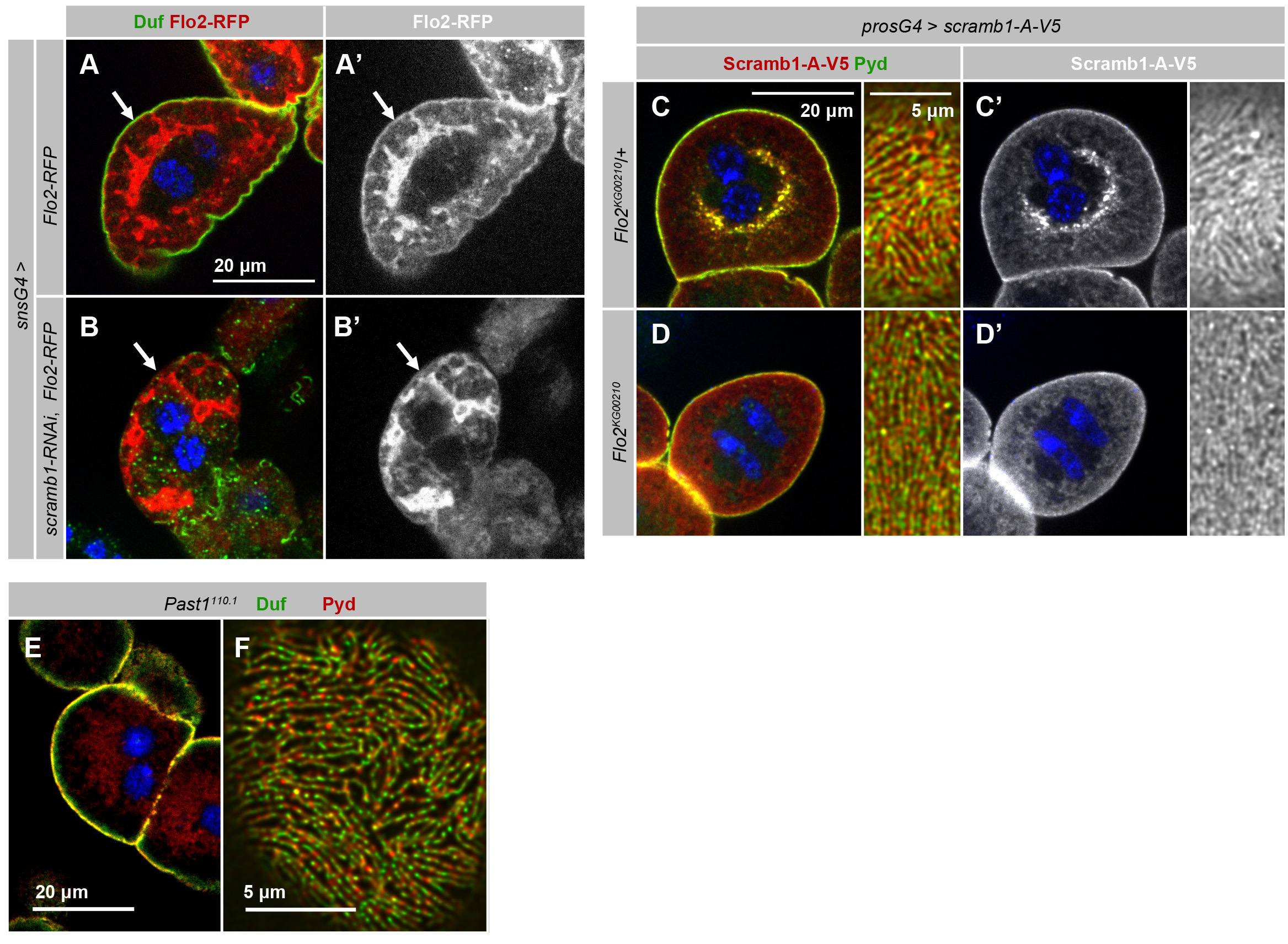

Supplement: Supplementary file 7 — Supplementary Material 7 [file 18_2024_5287_MOESM7_ESM.jpg]

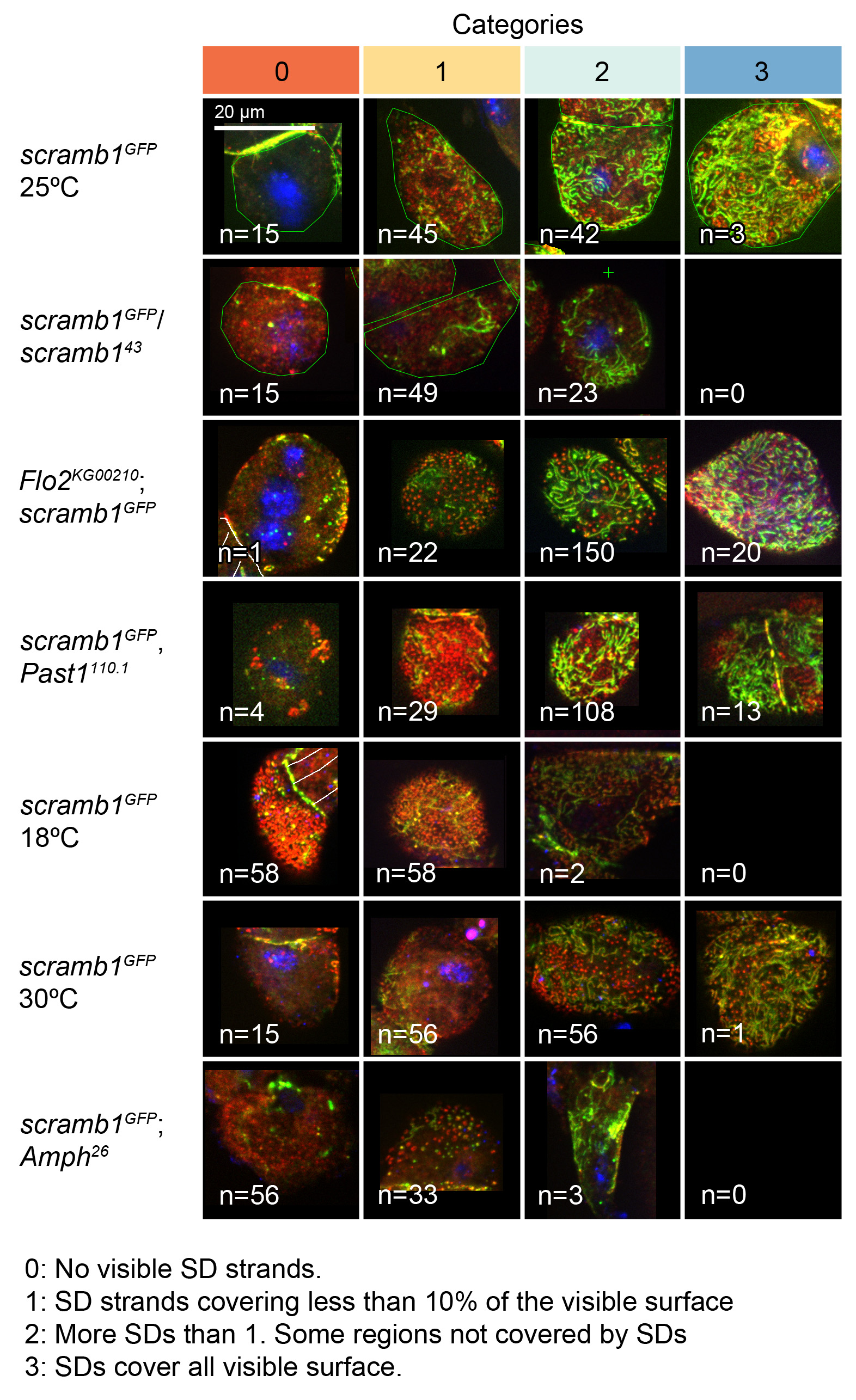

Supplement: Supplementary file 8 — Supplementary Material 8 [file 18_2024_5287_MOESM8_ESM.jpg]

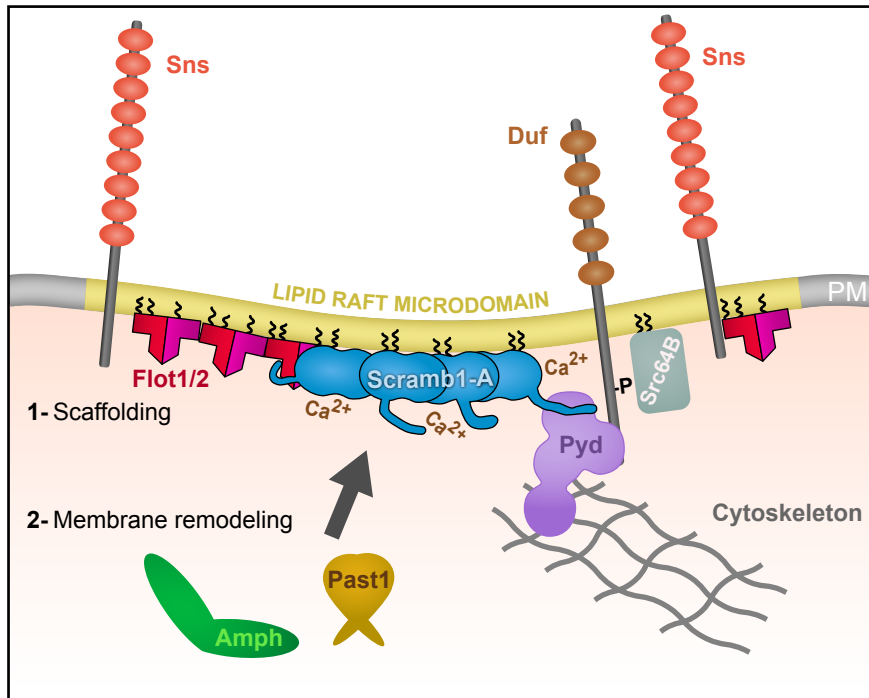

Supplement: Supplementary file 9 — Supplementary Material 9 [file 18_2024_5287_MOESM9_ESM.pdf]
