# Supplementary material for: Phospholipid scramblase 1: an essential component of the nephrocyte slit diaphragm
Source: Cell Mol Life Sci. 2024 Jun 15;81(1):261. doi: 10.1007/s00018-024-05287-z (PMC11335299; doi:10.1007/s00018-024-05287-z)
Supplement: Supplementary file 10 — Supplementary Material 10 [file 18_2024_5287_MOESM10_ESM.pdf]

Gene symbol	Gene name	Localization	Score
**ATPsynβ**	ATP synthase, β subunit	ER, nucleus	1209
**sesB**	stress-sensitive B	mitochondria	1113
**Phb1**	Prohibitin 1	mitochondria	809
**Hsc70-4**	Heat shock protein 70 cognate 4	cytosol	712
**Phb2**	Prohibitin 2	mitochondria	575
**Adh**	Alcohol dehydrogenase	cytosol	508
**Rack1**	Receptor of activated protein kinase C 1	cytosol	451
**Idh3a**	Isocitrate dehydrogenase 3a	mitochondria	451
**Ubi-p5E**	Ubiquitin-5E	cytosol	449
**Hsc70-3/BiP**	Heat shock protein 70 cognate 3	ER	362
**P5CS**	Δ^1-pyrroline-5-carboxylate synthase	mitochondria	362
**Idh3b**	Isocitrate dehydrogenase 3b	mitochondria	315
**l(2)efl**	lethal (2) essential for life	perinuclear, Z discs	277
**Mpcp2**	Mitochondrial phosphate carrier protein 2	mitochondria	260
**Idh3g**	Isocitrate dehydrogenase (NAD(+)) 3 non-catalytic subunit gamma	mitochondria	237
**eEF1α1**	eukaryotic translation elongation factor 1 alpha 1	cytosol	233
**Ldh**	Lactate dehydrogenase	mitochondria, cytosol	229
**sta**	stubarista	ribosome	215
**Gs2**	Glutamine synthetase 2	cytosol	206
**Pfk**	Phosphofructokinase	cytosol	205
**PCB**	Pyruvate carboxylase	mitochondria	204
**VhaAC39-1**	Vacuolar H^+ ATPase AC39 subunit 1	lysosome	191
**Hip14**	Huntingtin-interacting protein 14	Golgi, synapse	191
**Pp2A-29B**	Protein phosphatase 2A at 29B	cytosol	188
**CG11911**	SP103		179
**CG7255**	homologous to: solute carrier family 7 member 1-3	plasma membrane	168
**CG3902**	Orthologous to human ACADSB (acyl-CoA dehydrogenase short/branched chain)	mitochondria	158
**Mpcp1**	Mitochondrial phosphate carrier protein 1	mitochondria	148
**Vha68-2**	Vacuolar H^+ ATPase 68 kDa subunit 2	lysosome	143
**Vha100-2**	Vacuolar H^+ ATPase 100kD subunit 2	lysosome	136
**hfp**	half pint	cytosol	134
**CG5958**		cytosol	120
**Gapdh2**	Glyceraldehyde 3 phosphate dehydrogenase 2	cytosol	116
**Tsp42Ek**	Tetraspanin 42Ek	plasma membrane	113
**Past1**	Putative Achaete Scute Target 1	endosomes	111
**Tps1**	Trehalose-6-phosphate synthase 1	cytosol	105
**Argk1**	Arginine kinase 1	cytosol	94
**Eno**	Enolase	cytosol	94
**CCT6**	Chaperonin containing TCP1 subunit 6	cytosol	93
**ATPsynγ**	ATP synthase, γ subunit	mitochondria	92
**UQCR-C2**	Ubiquinol-cytochrome c reductase core protein 2	mitochondria	92
**wal**	walrus	mitochondria	89
**Vha55**	Vacuolar H^+-ATPase 55kD subunit	endosomes	88
**slf**	schlaff	plasma membrane	88
**Ih**	I_h channel	plasma membrane	86
**aralar1**	aralar1	mitochondria	83
**Sumo**	Small ubiquitin like modifier	nucleus, cytosol	79
**Ald1**	Aldolase 1	cytosol, Z disc, M band	78
**Hsp23**	Heat shock protein 23	cytosol	74
**CG8888**		plasma membrane	73
**mt:CoII**	mitochondrial Cytochrome c oxidase subunit II	mitochondria	73
**Flo2**	Flotillin 2	plasma membrane	73
**CG5853**		plasma membrane	69
**CG5177**		extracellular	65
**CG3164**		plasma membrane	65
**ScsβA**	Succinyl-coenzyme A synthetase β subunit, ADP-forming	mitochondria	65
**Pgi**	Phosphoglucose isomerase	cytosol	63
**Cand1**	Cullin-associated and neddylation-dissociated 1	cytosol	56
**Rpt6**	Regulatory particle triple-A ATPase 6	cytosol	55
**CG1907**		mitochondria	51
**whd**	withered	mitochondria	50
**Trap1**	Trap1	mitochondria	50
**Galt**	Galactose-1-phosphate uridylyltransferase	cytosol	49
**regucalcin**	regucalcin	extracellular	49
**Sfxn2**	Sideroflexin 2	mitochondria	48
**Gnmt**	Glycine N-methyltransferase	cytosol	47
**CG8036**		cytosol	44
**CG1572**		plasma membrane	44
**CCT1**	Chaperonin containing TCP1 subunit 1	cytosol	43
**eIF4G1**	eukaryotic translation initiation factor 4G1	cytosol	42
**Mdh2**	Malate dehydrogenase 2	mitochondria	41
**v**	vermilion		40

Table S1. List of proteins co-purifying with Scramb1-A-ProtA identified by mass spectrometry.

Only proteins absent in the control experiment, performed with empty matrix, are listed.
